# Saline nasal irrigation and gargling in COVID-19: a multidisciplinary review of effects on viral load, mucosal dynamics, and patient outcomes

**DOI:** 10.3389/fpubh.2023.1161881

**Published:** 2023-06-16

**Authors:** Suzy Huijghebaert, Shehzad Parviz, David Rabago, Amy Baxter, Uday Chatterjee, Farhan R. Khan, Cristoforo Fabbris, Konstantinos Poulas, Stephen Hsu

**Affiliations:** ^1^Non-Profit Research, Antwerp, Belgium; ^2^Medstar Health, Brooke Grove Rehabilitation Village, Sandy Spring, MD, United States; ^3^Infectious Disease, Adventist Healthcare, White Oak Medical Center, Silver Spring, MD, United States; ^4^Departments of Family and Community Medicine and Public Health Sciences, Penn State College of Medicine, Pennsylvania, PA, United States; ^5^Department of Emergency Medicine, Augusta University, Augusta, GA, United States; ^6^Department of Paediatric Surgery, Park Medical Research and Welfare Society, Kolkata, West Bengal, India; ^7^Department of Surgery, Aga Khan University, Karachi, Pakistan; ^8^Otolaryngology Unit, Treviso Hospital, Treviso, Italy; ^9^Department of Pharmacy, University of Patras, Patras, Greece; ^10^Department of Oral Biology, Augusta University, Augusta, GA, United States; ^11^Department of Oral Health and Diagnostic Sciences, Augusta University, Augusta, GA, United States

**Keywords:** saline, COVID-19, nasal irrigation, gargling, nebulization, nasal spray, respiratory infection

## Abstract

With unrelenting SARS-CoV-2 variants, additional COVID-19 mitigation strategies are needed. Oral and nasal saline irrigation (SI) is a traditional approach for respiratory infections/diseases. As a multidisciplinary network with expertise/experience with saline, we conducted a narrative review to examine mechanisms of action and clinical outcomes associated with nasal SI, gargling, spray, or nebulization in COVID-19. SI was found to reduce SARS-CoV-2 nasopharyngeal loads and hasten viral clearance. Other mechanisms may involve inhibition of viral replication, bioaerosol reduction, improved mucociliary clearance, modulation of ENaC, and neutrophil responses. Prophylaxis was documented adjunctive to personal protective equipment. COVID-19 patients experienced significant symptom relief, while overall data suggest lower hospitalization risk. We found no harm and hence recommend SI use, as safe, inexpensive, and easy-to-use hygiene measure, complementary to hand washing or mask-wearing. In view of mainly small studies, large well-controlled or surveillance studies can help to further validate the outcomes and to implement its use.

## Keypoints

- Oronasal saline irrigation is an inexpensive intervention to prevent and relieve common colds and upper respiratory infections.
- COVID-19 patients may also benefit, as saline irrigation of the nose and throat was found to reduce nasopharyngeal viral load, fasten viral clearance, and relieve symptoms of COVID-19.
- Overall, the small studies point to a potential benefit of saline irrigation on transmission, hospitalization, oxygen need, ICU admission, and/or mortality; confirmation in large trials is warranted.
- Mechanisms of saline include, apart from the rinse effect that can limit micro-aspiration of virus and secretions from the nasopharynx to deeper airways and lungs, also direct effects of iso- or mild hypertonic saline on SARS-CoV-2 replication (impairment of growth and fusion *in vitro*), mucosal hydration, mucociliary clearance, ENaC channel activity, and obstructive mucus/NET formation, all mechanisms that can help to reduce and prevent the development of more invasive severe disease.
- Oral and nasal saline rinsing can be considered for prevention and as an early intervention in SARS-CoV-2 infection, adjunctive to current standard protection including mask wear, distancing, and hand hygiene.
- Saline nebulization can be considered in patients who cannot do saline irrigation, or with lower respiratory tract signs or symptoms: The released bioaerosol does not contain live virus.

## 1. Introduction

In the ongoing COVID-19 pandemic, measures to limit the spread of SARS-CoV-2 have been a continuous and evolving challenge (1). Continuous mutations of its spike (S) protein circumvent effective vaccine and monoclonal antibody development (2, 3). New antiviral treatments are expensive and subject to side effects (4). Saline irrigation (SI) of the nose and throat is an inexpensive hygienic option that is proven effective for reducing the burden of other viral, bacterial, and acute or chronic respiratory illnesses (5–8) and has been empirically suggested as option for SARS-CoV-2 mitigation (9–12). Today, SI is also removed from the general WHO myth buster page (13, 14). While consensus guidance on its use has recently been proposed in China by a Chinese Rhinopathy Research Cooperation Group (15), published evidence for the utility of SI as adjunctive hygiene in COVID-19 has yet to be reviewed. We conducted a narrative review of this topic and present the clinical evidence and currently understood mechanisms for SI's efficacy in COVID-19 and propose recommendations for use and further evaluation.

## 2. Methods

Mixed methods were used for this descriptive review, generated with systematic search strategies, as described in the Supplementary Appendix Part A. The objectives of the reviewing process were to review the evidence for saline in COVID-19 by (1) evaluating clinical outcomes obtained with saline as (oro)nasal irrigation fluid or gargling in COVID-19 or SARS-CoV-2 infection and (2) exploring clinically relevant mechanisms of how these outcomes may be achieved—this with the objective to consolidate benefits and risks, as well as formulate recommendations. Nebulization was added as the search term to allow assessment of the harm–benefit balance of this procedure, often discouraged in COVID-19 (16).

**Figure 1:**
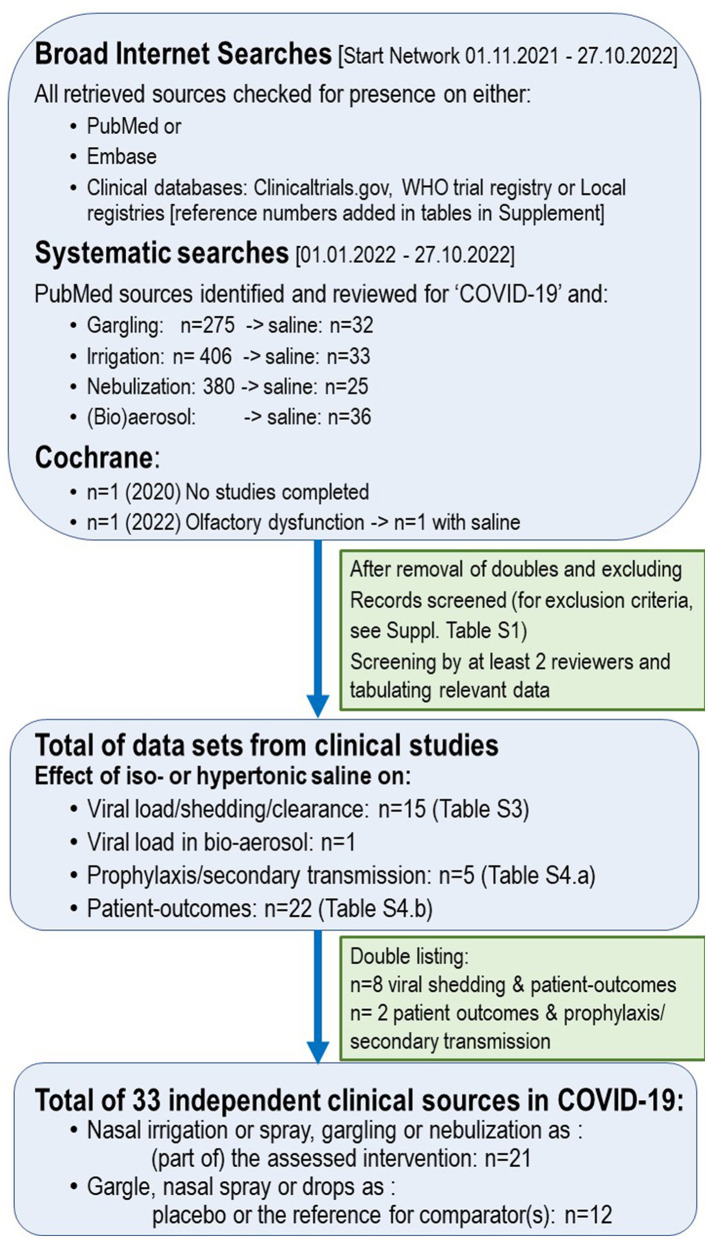
Search strategy—clinical data.

The search strategies for the clinical data and mechanisms of action are reported in Supplementary Appendix Part A.1. p. 02–05, (Supplementary Table S1) and Supplementary Appendix Part A.2. p. 10–11 (Supplementary Table S2), respectively. With the literature on saline being abundant, a stricter search strategy was conducted for clinical reports of relevance on PubMed from 1 January 2022 to 27 October 2022 (Figure 1). Initial screening of clinicaltrials.gov and WHO trial databases had revealed that saline was not the subject of a large public or developmental program, while it was often also serving as a reference or placebo, which explains the substantial heterogeneity of study designs. Therefore, studies were eligible, irrespective of whether saline (irrigation, mouth rinse, gargling, spray/drop, or nebulization) was the subject or the reference/placebo of the study. In view of the exploratory nature of this review, additional searches on the Internet and PubMed were performed, with the use of additional search terms, described in Supplementary Appendix, p. 03, such as a clinical trial or clinical study, nasal sprays or drops, or using the protocol numbers from trial databases, as to retrieve eventual results. The material was progressively compiled. Details on reasons for rejection of sources are listed in Supplementary Appendix, p. 03–06 and p. 10, respectively. Most studies were small. If a bias was identified, this was noted in the tables and/or discussed in the text. In total, 33 clinical studies or reports were eligible and tabulated, which are stratified (Supplementary Appendix, p. 06, 07) by the effects of saline on viral load and/or clearance (Supplementary Table S3, p. 11) and/or effects on clinical outcomes (Supplementary Table S4, p. 17). Rates of hospitalization and risks (intensive care uptake, pneumonia severity scores, ventilation, and mortality) were compiled separately in Supplementary Table S5, p. 30. Grading, pooling, or meta-analysis of outcomes was not performed due to often small-sized (local) studies, substantial heterogeneity in study design, disease severity and outcome parameters, and/or poor or limited data reporting.

**Figure 2:**
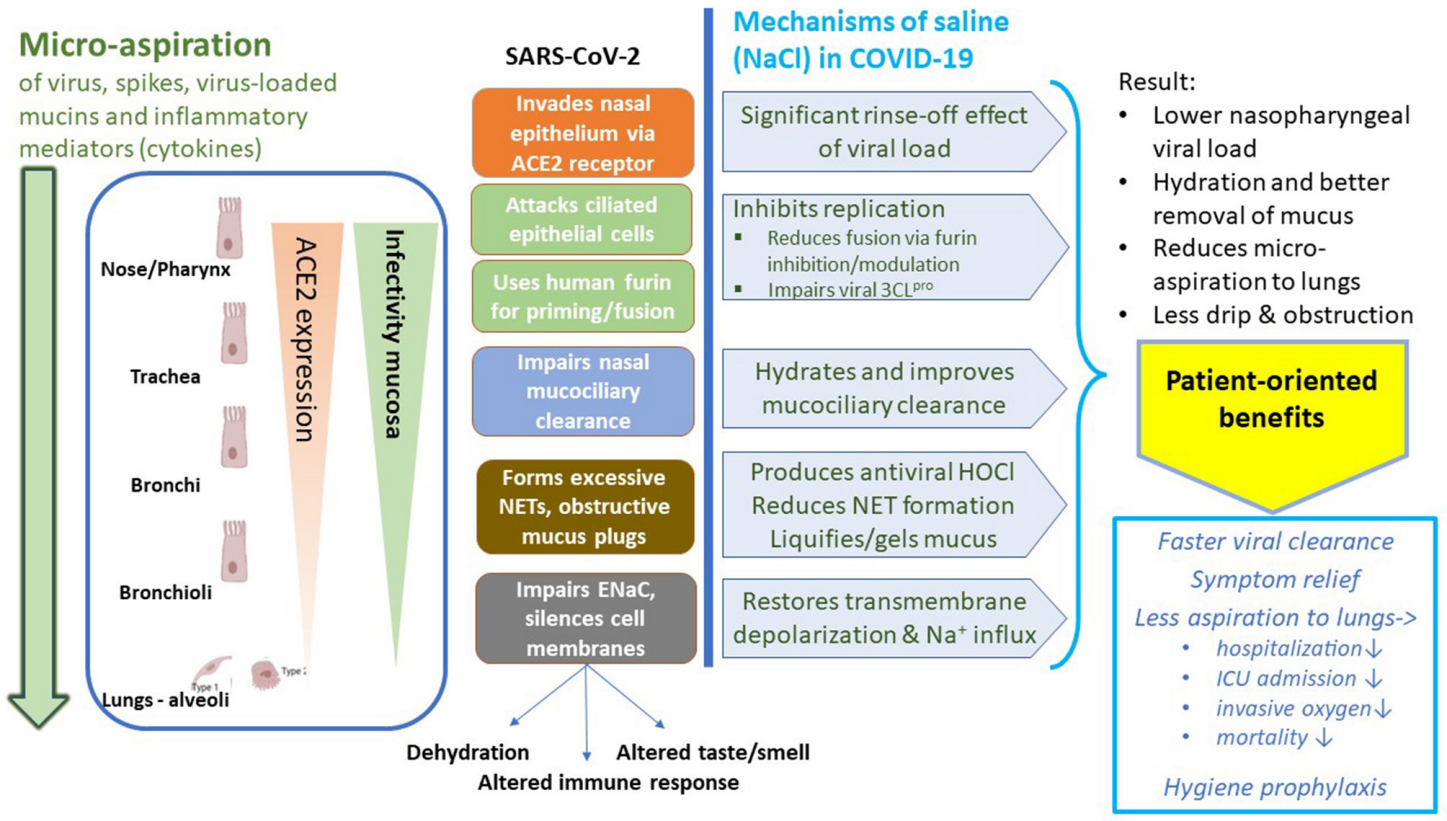
Mechanisms of action of saline relevant to COVID-19. ACE2, angiotensin-converting enzyme 2; NET, neutrophil extracellular trap; ENaC, epithelial sodium channel; HOCl, hypochlorous acid formed through neutrophil myeloperoxidase activity in the presence of NaCl.

For the mechanisms of action of saline of relevance to COVID-19, initial search strategies (Supplementary Appendix, p. 10) addressed the effect(s) of saline on SARS-CoV-2 viral load (aspiration), replication, exhaled bioaerosol, ENaC channel activity, hydration and mucociliary clearance, neutrophil response, and hypochlorous (HOCl) formation. Yet, mechanisms were also examined from a broader translational view by the Network. As the COVID-19 literature is extensive, sources were limited to one (if consistent) or the most representative articles or reviews (if multiple sources or reviews). The mechanisms addressed and found in support of SI are summarized in Figure 2.

The findings of the review process were compiled according to (A) the major mechanism documented clinically for saline irrigation (viral load reduction) and its relevance to transmission, (B) additional mechanisms that can be effectuated by saline mucosal irrigation, and that may be protective in the initial stage of COVID-19 (sodium chloride (NaCl) homeostasis and innate immune response), and (C) clinical outcomes of studies in COVID-19. Clinical benefits, risks, lessons learned, recommendations, and conclusion were formulated and reflect the final consensus in January 2023, after evaluation and discussion of the benefit–harm balance, based on the consolidated findings from the tabulated data.

## 3. Results

### 3.1. Viral load reduction and transmission

#### 3.1.1. SARS-CoV-2 pathophysiology: rationale for viral load reduction

Similarly as for common colds (8), the main rationale proposed for using saline irrigation is to rinse the nasal and throat mucosa, removing SARS-CoV-2 and limiting the viral load from these tissues.

Nasal multiciliated cells are primary targets for the entry of SARS-CoV-2 (17), which after replication are released at their apical side (18), allowing rapid reinfection and progressive epithelial damage, with a spread from the naso- and oropharynx to the deeper throat and airways. The infection initially starts almost exclusively in the ciliated epithelia of the nose (19), while micro-aspiration of virus, sputum, mucins, and inflammatory secretions from the oral and nasal cavities is increasingly recognized as the main mechanism for infecting deeper airways and lungs (Figure 2) (20–22). This pathway in SARS-CoV-2 pathophysiology is supported by the findings of (upregulated) expression patterns of SARS-CoV-2 entry genes (such as ACE2 and TMPRRS2) and broad nasal, buccal, and blood gene expression analysis, the absence of alveolar macrophages and alveolar invasion at onset of the disease, the difficulty to infect human lung tissues with SARS-CoV-2 *in vitro* (in contrast to nasal and *in vitro* host cells), and the progressively observed immune gene upregulation (20–25). In addition, mucin secretions are abnormal and become upregulated, as well as nasal inflammatory cytokines (26, 27). Mucins often present as viscous plugs obstructing the trachea and bronchi (see also Section 3.2.2.). The highest viral loads are detected in sputum, followed by nasopharynx, while these are usually lower in saliva (28–30). Moreover, development of viral SARS-CoV-2 RNA shedding and bronchopneumonia was found to be dose-dependent in response to the dose of virus intranasally delivered, supporting the concept that rinsing off virus in an early stage may reduce disease development and severity (31). To further illustrate the importance of the removal of aerosolized secretions, it is important to note that elements of the virus itself, such as the released S proteins, can generate inflammation and tissue damage in the picomolar range, as well as bind multiple innate immune receptors, contributing to the disturbed local immune response (32, 33)]. An *in vitro* study showed that endothelial cells are normally not invaded by SARS-CoV-2; following viral replication, however, epithelial released cytokines and components induced during the infection affected the neighboring endothelial cells, resulting in damage of the epithelial/endothelial barrier function and causing the viral dissemination (34). Observations that the cytokine levels in sputum, and not serum, are predictive for lung damage and prognosis of severe COVID-19 (35), and that mucus surprisingly releases finer aerosol particles the more its viscoelasticity decreases (36) (dry plugs common in COVID-19), support further the concept of micro-aspiration of virus and infected bioaerosol to the deeper airways and lungs.

Micro-aspiration of virus and secretions from the oronasal cavity not only allows progressive viral invasion of deeper airways and lungs causing COVID-19 pneumonia but also may contribute to severe co-infected pneumonia: Viral, bacterial, and fungal secondary infections are common in SARS-CoV-2 patients, and viruses, bacteria, and fungi were frequently found in the plugs removed from the trachea and bronchi during bronchial/tracheal lavage (37–40). Studies on microbiota identified depletion of beneficial commensals and an upsurge of opportunistic pathogens, also in the oral and nasopharyngeal cavity (41).

Higher SARS-CoV-2 viral loads and longer virus shedding have been associated with disease severity and disease progression, as well as with transmission (42–44). Hence, their reduction may help to avert the infection, reduce micro-aspiration of the virus to the lower respiratory tract, and prevent the progression to a more severe disease state. Earlier studies in other respiratory conditions have shown that nasal SI helps to remove pathogens, allergens, debris, and secretions from the nasal cavity and reduce sinonasal and respiratory infections (6–8, 10, 45), while respecting the normal sinonasal commensal microbiome (46). SI is, therefore, a reasonable choice to reduce viral loads and micro-aspiration during COVID-19 and potentially help prevent the development of pneumonia and acute respiratory distress syndrome (ARDS).

As both the nasal and oropharyngeal cavity may become infected, and so may lead to both micro-aspiration of the virus to the lungs and exhaled infectious bioaerosol, and so to airborne transmission, (also via saliva) (47), the effects of nasal irrigation, gargling and nebulization of saline on viral loads, bioaerosol formation, transmission, and prophylaxis are considered in this section.

#### 3.1.2. Rinse effect reducing viral loads

Fifteen studies were identified, reporting on the effect of saline gargles (*n* = 4) and SI (*n* = 9) or nasal spray (*n* = 2) on viral load or clearance to PCR-negative status (Supplementary Table S3, p. 11). Symptomatic and asymptomatic patients, different saline concentrations (0.9–5%) and schedules, routes (nasal, gargling, and combined), and methods of SI (neti pot, spray or pressurized bottle, or lavage system) were evaluated (48–62).

**Figure 3:**
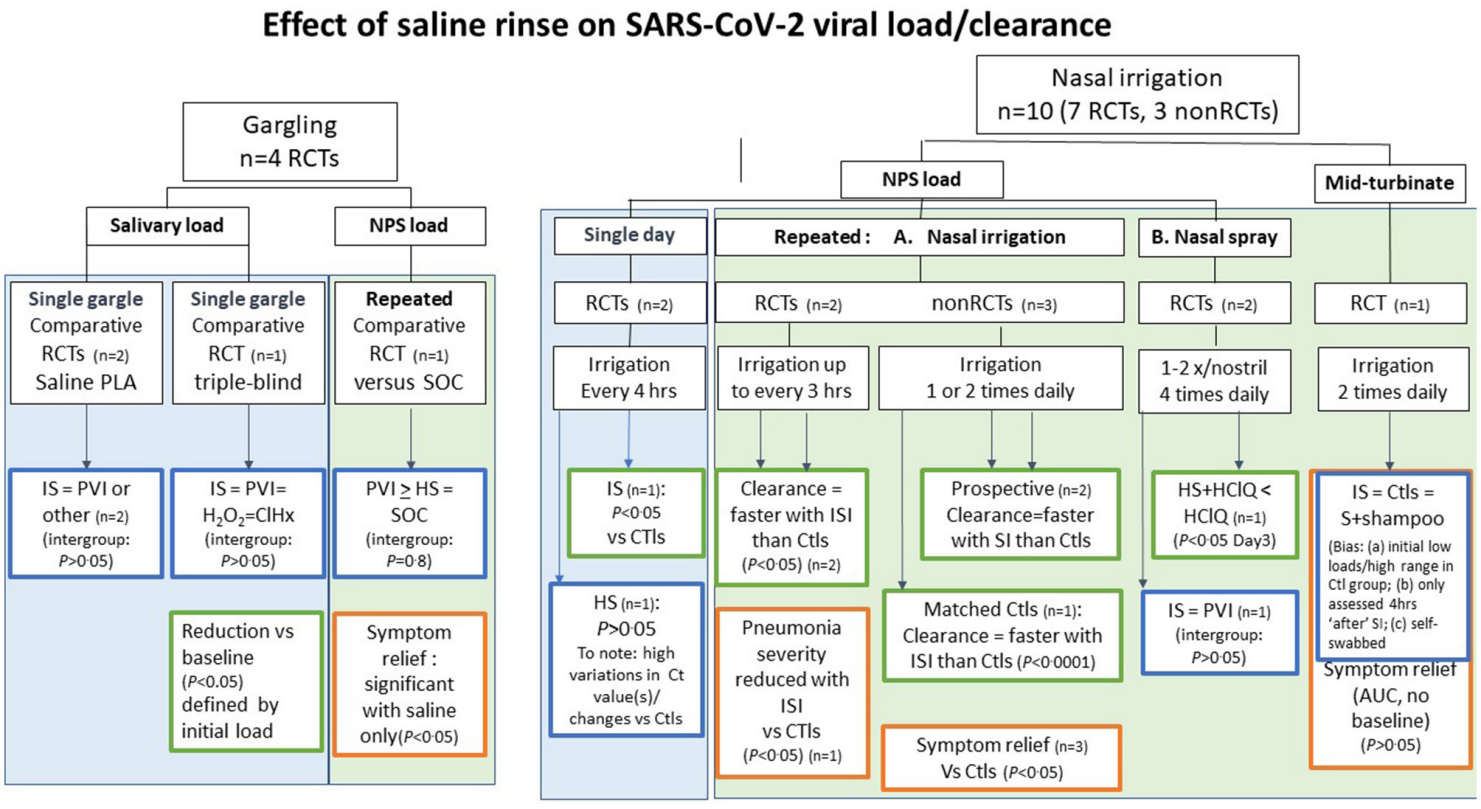
Clinical studies or reports of saline rinse or SI on SARS-CoV-2 viral load and clearance, stratified by method (gargling or nasal irrigation/spray) and the type of viral load (salivary, nasopharyngeal swabbed (NPS), or mid-turbinate): schematic overview of benefit–risk balance assessment. RTC, randomized clinical trial; non-RCT, non-randomized clinical trial; *n*, number of studies; CTls, controls; IS, isotonic saline (ISI, isotonic saline irrigation); HT, hypertonic saline; HClQ, hydroxychloroquine; PVI, polyvidone iodine; H_2_O_2_, hydrogen peroxide; ClHx, chlorhexidine; AUC, area under the curve of cumulative total symptom scores. Significant difference vs. controls or comparator: *P* < 0.05; non-significant *P* > 0.05.

The studies and their outcomes are schematically represented by salivary, nasopharyngeal, and mid-turbinate viral load testing, and their randomized (RCT) or non-randomized controlled trial (non-RCT) designs are shown in Figure 3. Overall, studies in COVID-19 patients report that single-day and repeated SI reduces nasopharyngeal viral loads.

##### 3.1.2.1. Single gargle studies (n = 3)

Two studies evaluating the effect of a single gargle (after 5 or 30 min) of an antiseptic mouth rinse vs. a short 30-s 10–20 mL saline gargle (control) did not find significant intergroup differences in mean salivary viral load reductions between saline and the antiseptic mouth rinses (49, 50). In another study, the median salivary viral load wa s reduced by 89% at 15 min after gargling 20 mL of saline for 60 s (90% after 45 min) vs. baseline. The median changes were similar for the three antiseptic comparators varying between 61–89% and 70–97%, respectively, while mean reductions (25–75%) highly fluctuated in values (*P* > 0.05 upon intergroup comparison) (48). There was, however, a significant correlation between baseline viral load and reduction at 15 min (*P* = 0.0073) and persistence at 45 min (*P* = 0.0087) for all mouth rinses tested. In cases where the viral load was <10^4^/mL copies, a 100% reduction was obtained (48).

##### 3.1.2.2. Single-day SI studies

Mean nasopharyngeal viral load in hospitalized COVID-19 pneumonia patients was reduced by 17.3% (median 23.6%) with hypertonic saline after three nasal SIs performed within 6 h while not among the small control group (not reaching significance) (52), while a larger randomized controlled study with 0.9% SI, 10 mL in each nostril every 4 h for 16 h, found a significant reduction in nasopharyngeal viral load at 24 h vs. the control group (*P* = 0.005) (51).

##### 3.1.2.3. Repeated SI studies

After 3–10 days of oronasal SI, fast(er) viral clearance was observed compared with controls in most studies (51, 54–59). Start of SI immediately after testing cleared the virus in 91% of patients on Day 10 vs. 28% in matched controls not receiving SI (*P* < 0.0001) (54). Even a delayed start of SI (7 days after testing) also favored its use in a prospective non-RCT (55).

In three RCTs, it was shown that SI or nasal spray added to standard of care (SOC) also cleared viral loads faster in more severe (56) or hospitalized cases (58, 59). In a dose-finding study done in COVID-19 outpatients with pneumonia without ARDS, regular SI (20 mL every 3 h) led to more patients becoming PCR-negative on Day 5 than controls (68 vs. 25%; *P* < 0.05) (56). As further discussed under Section 3.3.3, this was associated with improvement in SARS-COV-2 pneumonia. Nasal SI resulted in significant earlier clearance in hospitalized patients for Omicron infection (*P* < 0.05) (59). Hypertonic nasal spray use was found to reduce significantly the nasopharyngeal viral load during the initial treatment days when used add-on to hydroxychloroquine vs. hydroxychloroquine alone (*P* = 0.019) (58).

##### 3.1.2.4. Salivary and mid-turbinate loads

Salivary or mid-turbinate viral load reductions, obtained with saline nasal spray or SI, were not significantly different between saline and antiseptic comparators (mouth rinse or nasal spray containing polyvidone iodine, chlorhexidine, or detergent) (48–50, 53, 60, 61). Short (15 s) hypertonic saline gargling three times daily did not render mild patients getting faster negative in the PCR test (sampled by nasal and oropharyngeal swabbing) than their controls (median 9 days), despite significant symptom recovery with saline only (Day 4, *P* < 0.04) (53). These data suggest that oronasal SI or the combination of nasal rinse and gargling may achieve the highest effect.

#### 3.1.3. Viral replication inhibition *in vitro*

Saline (0.9–1.3% = 150–200 mM) has been reported to reduce viral replication by 50–98% *in vitro*, thereby reintroducing voltage-gated depolarization in the infected host cells (63). These concentrations also reduce the activity of furin, a human protease, hijacked by SARS-CoV-2 for cleaving the unique furin cleavage site in its spike, thereby priming the spikes for cell fusion (64), and involved in its virulence (65). Furin is highly expressed in salivary glands and present in saliva, and it has been proposed to induce virus pre-activation, which could favor prompt spread through saliva droplets of more infectious virions than those through sneezing and coughing (66, 67). Saline at these salt concentrations also impairs 3CLpro (Mpro), a SARS-CoV-2 protease regulating replication (68). These modest effects may be relevant to combining nasal SI with protease inhibitors to reduce relapse with latter antivirals (62, 69).

#### 3.1.4. Effects on exhaled viral load/bioaerosol

As reviewed (70), NaCl has unique ionic properties leading to droplet/vesicle aggregation, optimal viscosity, and properties of mucus and reduced bioaerosol. Faster hygroscopic growth of iso-/hypertonic bioaerosol and mucus droplets due to saline leads to earlier deposition in nasopharynx/airways, while these particles will be removed continuously from the airways via the mucociliary clearance and be swallowed, and so limits penetration of pathogens in the deeper airway and lungs (71–73). In line with these unique ionic properties of saline, nebulizing saline was found to reduce exhaled droplet numbers in bioaerosol superspreaders (74), while in hospitalized COVID-19 patients, the continuously sampled bioaerosol during 0.9% saline nebulization was found to be devoid of SARS-CoV-2 RNA or live virus: In contrast, nebulization of viral culture, equivalent in titer to the sampled nasopharyngeal load of these patients, resulted in positive bioaerosol samples (75). So, there is no evidence of dissemination by the aerosolizing/nebulizing procedure. It is interesting to note that in a study of 12 hospitalized qPCR-positive patients, nasopharyngeal viral loads (sampled following a 20-mL saline gargle to generate baseline values before H_2_O_2_ assessment) were low, with only five in 12 showing loads expected to contain viable virus, while intriguingly upon culturing, only one viable out of the five (76). The relevance of these observations deserves further study, whether due to the sampling method, or saline rinse effects [such as diluting and/or aggregating virus, reducing furin-mediated priming, and/or deactivating virus via salivary myeloperoxidase (see Section 3.2.3)]. Facial mask impregnation with salt/saline has been found to lead to better filtration or deactivation of virus (70, 77).

#### 3.1.5. Reduced transmission and prophylaxis

Overall, publications on the prophylactic use of SI in COVID-19 are limited so far (*n* = 5). One study relates to SARS-CoV transmission (78), and three sources were related to SASR-CoV-2 transmission, during the initial (79), Alpha-Delta (80), and omicron wave (59), while one more experience was solely reported in the press (therefore not tabulated) (81). One randomized study used local saline brands containing minimal amounts of chlorine (nose rinse spray and mouthwash) in healthcare workers (HCWs) during the first COVID-19 wave (82) (For details, see Supplementary Table S4, p. 17).

An earlier randomized trial assessing SI for common colds reported a 35% reduction in household transmission of respiratory viruses, including human coronavirus, compared with controls (8). In line with this controlled study, a prospective study of SI in COVID-19 patients found limited household contacts testing positive after SI (12.7%) (80); expected rates for within-household transmission average 18.8–27.0% (range 4.6–90%; for the original variant) (83, 84) and 15–35% according to a large UK study (Alpha variants are 50% more infectious than wild-type while 35% less infectious than Delta) (85)–(relevant to this study, run 24 September−21 December 2020).

Successful prophylaxis has been reported in HCWs using SI adjunctive to personal protective equipment (PPE), during initial (79, 82), Delta (81), and Omicron waves (59). This beneficial effect of nasal rinsing was already observed during the first SARS-CoV wave (78). A significant reduction was found in an initial randomized prophylactic study with electrolyzed SI (nose rinses and mouthwashes), resulting in 12% HCWs becoming infected with saline prophylaxis and 12% without (*P* < 0.05) (82). An Indian press report reported similar protection rates during the Delta wave (no study details available): 1% of HCWs using daily SI became PCR-positive compared with 10% in the wards not using SI (81). A COVID-19 designated hospital in China recently reported zero Omicron infections among HCWs in a large COVID-19 designated hospital in Shenzhen (1,930 COVID-19 patients admitted during Omicron peak), all obliged to use daily SI add-on to strict PPE wear (59).

Indirect support for SI and nebulization in reducing transmission also comes from an ecological regression analysis, finding that highly populated coastal areas with high concentrations of atmospheric salt recorded significantly fewer COVID-19 infections and deaths per capita compared to inland regions by 25–30% (*P* < 0.05) (86).

### 3.2. Mucosal NaCl homeostasis and innate immune response

#### 3.2.1. Modulation of ion channels and homeostasis in the mucosa

A commonly overlooked effect of SARS-CoV-2 infection is the impairment of ENaC (sodium channel) activity by SARS-CoV-2 and its S and E proteins, likely due to the virus competing for and so hijacking furin, a ubiquitous human protease activating ENaC (63, 87). They silence the cell membranes, eliminating voltage-gated transmembrane potentials and depolarization, so impairing Na^+^-influx and fluid reabsorption (63, 87). This leads to a complex dysregulation of many processes requiring salt gradients or ion transport, some of which are discussed below, others are beyond the scope of this article: For instance, ENaC is the driving regulator of airway surface liquid and mucosal hydration, ciliary motion, mucus transport, and reabsorption of airway and alveolar secretions; beyond the respiratory tract, ENaC also (co)regulates taste buds, ACE2, systemic hemodynamic, and immune responses (88–90). As saline re-introduces depolarizations and so restores Na^+^-influx in infected cells (63), SI may help to modulate or limit ENaC-associated dysfunctional processes in the respiratory mucosa.

#### 3.2.2. Improved mucociliary clearance, mucus, and cough clearance

SARS-CoV-2 infection has been shown to impair MMC in SARS-CoV-infection (70, 91), which often causes nasal obstruction and dry cough (92). Established actions of iso/hypertonic SI in other respiratory diseases include hydration of the periciliary layer and improving MMC and cough clearance (70). These properties may contribute to faster SARS-CoV-2 clearance (see Section 3.1.2.). Saline profoundly changes the viscosity/elasticity of mucus and adhesion/cohesion of mucins, both hydrating and gelling/ thickening these toward easier transportable mucus (93–95). Moreover, the mucus production, stimulated by interferon-aryl hydrocarbon receptor signaling in SARS-CoV-2 infection, would trigger the hypoxia of COVID-19 (96). As mucus plugs (often combined with neutrophil extracellular traps (NETs) in COVID-19, see Section 3.2.3.) create local hypoxic environments that can contribute to enhanced secretion of destructive neutrophil granule contents (97), mucus removal by early SI may help to reduce and prevent nasal and airway obstruction and its deleterious consequences. Postnasal drip, nasal obstruction, cough, and sneeze are relieved by SI in mild-to-moderate COVID-19 (see Section 3.3.1.).

#### 3.2.3. Dampening neutrophil elastase activity

One of the altered immune responses in COVID-19 involves excessive neutrophil activity and NET formation, which is already observed (also nasally) early in the disease (98) and has been implicated in the development of acute lung injury, the induction of the SARS-CoV-2-induced cytokine storm, ARDS, and immuno-thrombosis (99, 100). NETs are composed of decondensed chromatin fibers coated with antimicrobial proteins, such as myeloperoxidase (MPO) and neutrophil elastase, aimed at capturing and neutralizing pathogens to prevent microbial dissemination (99). However, SARS-CoV-2 develops more invasive cell infection and higher pro-inflammatory responses in the presence of neutrophils *in vitro* (101), while the complex actions of MPO and elastase are thought to contribute to the inflammatory damage in COVID-19 (70, 99, 100, 102). SI may help to clear the adhesive mucus/NETs through the simple action of rinsing the mucosal surfaces, as evidenced by the therapeutic plug removal with saline bronchial lavage or washing in COVID-19 patients (37, 103, 104). Other relevant mechanisms that have been observed with hypertonic SI (>200 mM NaCl; 150 mM = isotonic) in respiratory disease include lowered NET formation and decreases in neutrophil elastase activity, pro-inflammatory cytokines, and reactive oxygen species (ROS) (NaCl ion-specific effects) (105–107).

#### 3.2.4. Local virucidal hypochlorous acid production

Moreover, NaCl has been shown to inhibit the replication of enveloped and non-enveloped DNA and RNA virus *in vitro*, via the production of virucidal hypochlorous acid (HOCl) by neutrophil phagosomes—considered to be an innate antiviral immune response and involving myeloperoxidase, thereby also providing a mechanism for oxidative NET-mediated microbial killing (70, 108–110). HOCl also controls neutrophil elastase activity and NET release (109, 111), so representing additional mechanisms by which saline may act to limit the illness progression. The role of this innate immune process in the inactivation of the virus [a study finding low infectivity of saline gargles from COVID-19 patients (76)] deserves further study, as myeloperoxidase activity is markedly increased in saliva and lungs of SARS-CoV-2 infected patients (112).

### 3.3. Clinical benefits of SI in COVID-19

**Figure 4:**
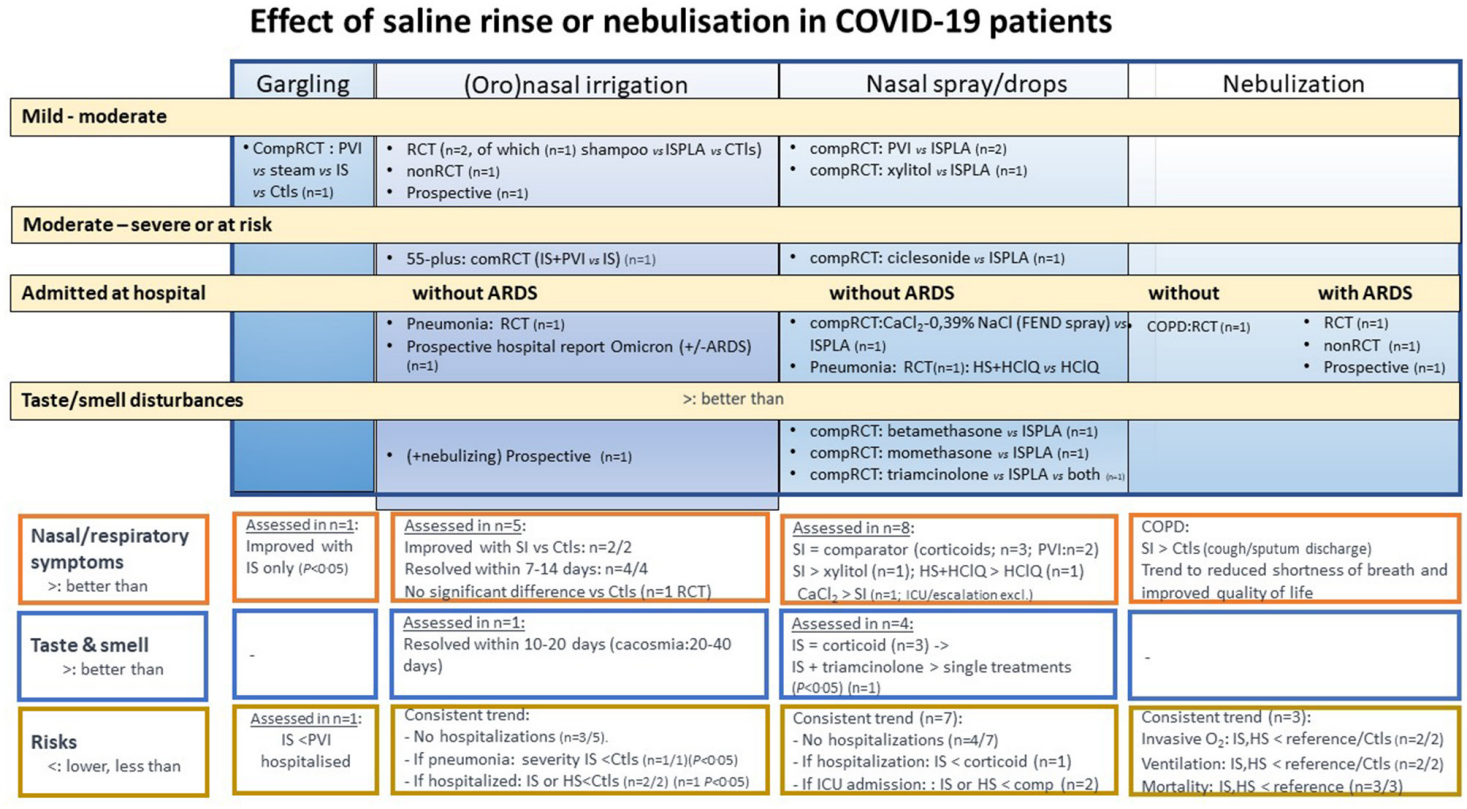
Effect of saline rinse or nebulization in COVID-19 patients, stratified by disease severity and type of rinse/nebulization: schematic overview of benefit–risk balance assessment. See also Figure 3; comp, comparative; O2, oxygen; IS, isotonic saline; ISPLA, isotonic saline as placebo; HS, hypertonic saline; ARDS, acute respiratory distress syndrome; COPD, chronic obstructive pulmonary disease. Meaning of symbols: **(A)** respiratory symptoms (orange framed outcome) and **(B)** taste/smell disturbances (blue framed outcomes): “=”, effect comparable between treatment groups; “>”, generally better outcome with saline than with Ctls or comparator; “<”, comparator doing better than saline placebo; **(C)** risks: <, rate or severity outcome lower with saline than Ctls, comparator or reference population.

Twenty-one eligible clinical (mainly small) studies were identified through PubMed and broad Internet searches (some reviewed for viral load under Section 3.1.2) (53–62, 80, 92, 113–124). They are listed in Supplementary Table S4, p. 17–29. As illustrated in Figure 4, these studies used ample-volume (oro)nasal SI (*n* = 8), repetitive saline sprays or drops (sometimes as placebo) (*n* = 8), gargling (*n* = 1) or nebulization (*n* = 3), spanning various study designs, disease severity, irrigants or nasal spray/drop comparators, and outcomes. As many studies were small and study samples were not powered to detect differences in outcomes on COVID-19-related risks, such as pneumonia, hospitalization, ICU admission, invasive oxygen need/ventilation, and mortality, the latter results are consolidated in Supplementary Table S5, p. 30.

#### 3.3.1. Respiratory symptom reduction

In a non-randomized case-controlled study, newly diagnosed COVID-19 patients, starting immediately isotonic SI once daily after PCR testing, experienced significantly more nasal symptom relief than controls (*p* < 0.05) (54). In addition, other studies comparing to controls have reported significant symptom recovery, reduction of postnasal drip, nasal obstruction, cough, or sneeze (*p* < 0.05) (53, 113, 114, 116, 121).

In a prospective study, PCR-positive patients older than 55 years (mean age 65 years), mostly with high BMI, were assigned to two different pressurized SI techniques (80). COVID-19 symptoms (loss of smell or taste, fatigue, fever >100.4°F, chills, muscle aches, runny nose, new onset (or worsening of chronic) cough, shortness of breath, nausea or vomiting, headache, abdominal pain, and diarrhea) were resolved after 14 days of SI in 63 of 71 participants (one hospitalization, see Section 3.3.3); symptom resolution was more likely for those reporting twice daily SI than once (*P* = 0.0032) (80).

Studies with nasal spray or drops comparing symptom relief with saline (as placebo) and various treatments (polyvidone iodine, calcium chloride, and ciclesonide) have not reported the superiority of SI, though they have shown symptomatic improvement consistent with the comparators (see Supplementary Table S4, p. 20^_^22) (53, 60, 80, 92, 115).

#### 3.3.2. Smell and taste recovery

Symptoms of smell or taste dysfunction in COVID-19 patients may take 10–40 days to resolve with SI (54, 61, 80, 113, 116–120). For specific studies in COVID-19 patients with smell and taste disturbances, see Supplementary Table S4, p. 23 (studies in long-COVID-19 patients were excluded). Overall SI and nasal steroids were found to perform equally well (no consistent statistically significant differences) (118–120). Yet, a significantly faster resolution was obtained with the combination of SI and triamcinolone, a finding that needs confirmation in larger clinical studies, while at best also evaluating the impact of frequency of SI (120).

#### 3.3.3. Hospitalization and intensive care risks

##### 3.3.3.1. SI or spray use

Although not set as primary outcomes, except for two studies (80, 113), many studies using SI or saline spray reported on the rate of hospitalizations, some ICU outcomes, and mortality. Overall, the studies were too small to detect significant differences. For tabulation of these outcomes, see Supplementary Table S5, p. 30.

If hospitalizations were observed, rates were generally lower with the saline formulations than with controls or comparators. Relevant to patients aged 55 or more years, Baxter et al., assessing hospitalization as a primary parameter, recorded one hospitalization [1/78 (1.3%)] (no deaths) with isotonic SI in this age group with high BMI: These rates were 9.5% (plus 1.5% deaths) in a nested control population over 50 years, over the same period (80). Another study in COVID-19 patients during the first wave found unexplained high hospitalization rates in both groups, yet lower with SI users, but not significantly reduced vs. controls (*P* = 0.66), while there was no high adherence to the rinse procedure (113).

In line with faster clearance of viral loads and so reduced risk of micro-aspiration from nose to lungs (see Section 3.1.1.), patients with COVID-19 pneumonia (without ARDS) experienced a significant reduction in computed tomogram-evaluated pneumonia severity score after 5 days of isotonic SI compared with controls (*P* < 0.05*)*; also the percent of patients worsening (8.8%) was lower when compared with controls (36.4%; *P* = 0.028) (56). In line with these results, another randomized study of isotonic SI, initiated in hospitalized COVID-19 patients and leading to faster viral clearance Day 2 (*P* < 0.02), suggests that rapid initiation of SI following a positive PCR test may help to lower subsequent need for oxygen administration, ICU admission, and mortality, although the differences did not reach statistical significance between the small patient groups (*n* = 25 per group), and a potential bias may have incurred by the average older age of the control group (51).

##### 3.3.3.2. Nebulization

Two prospective and one randomized hospital studies used nebulized saline adjunctive to SOC (122–124). In patients with ARDS, saline nebulization (+ibuprofenate) (123) was associated with improved respiratory rate (123), low need for invasive oxygenation or intubation (122, 123), and low(er) mortality (compared to controls) (123, 124). These findings are in line with observations in patients with bronchiolitis and other respiratory diseases, in whom iso- or hypertonic saline has been shown to improve oxygen saturation (125, 126).

In a four-arm study in ventilated patients with ARDS comparing two solutions vs. saline 0.9% and controls (twice daily), methicillin-resistant *Staphylococcus aureus* incidence significantly decreased with the nebulizing treatments vs. controls (12% for saline vs. 34.6% among controls; *P* < 0.05); mortality was lowest among the patients receiving nebulized saline (40.5 vs. 59.6% among controls; not significant) (124).

In line with the findings on bioaerosol, following saline nebulization in hospitalized COVID-19 patients (75) (reported in Section 3.1.4), nebulization was reported not to lead to transmission or spread of the virus among HCWs (122).

In conclusion, studies in COVID-19 patients have consistently reported safe use and outcomes favoring SI use in COVID-19, whether it was used as sole intervention initiated immediately after positive PCR testing in mild-to-moderate COVID-19, or as part of the patient treatment protocol adjunctive to SOC in an ambulatory setting or upon hospitalization. The results require confirmation in large, randomized studies.

### 3.4. Patient satisfaction and risk

This review found that SI is accepted and well-tolerated in all studies reported. Nasal burning or discomfort was reported consistent with other reports and clinical practice. These can often be ameliorated by reducing salinity and technique adjustment. Epistaxis is rare and was not reported in reviewed studies, except in older individuals (80). Nasal SI requires the use of clean water, as rare infection, and death with *N. fowleri* has been reported (127).

Concerns for SI affecting the blood pressure adversely have not proven to be a problem, as has been evidenced in patients with chronic lung disease, also at hypertonic concentration (128, 129). Patients should be advised not to swallow saline drip or gargles but to spit these out safely in a sink or receptacle.

Daily use of repeated SI may show lower compliance in vulnerable patients, such as those admitted in care homes with COVID-19. This challenge can be overcome by motivating home residents and staff to apply SI and survey the significant reduction in respiratory complications (130). SI materials (Neti pot and squeeze bottle) should be cleaned daily. Routine precautions for limiting the COVID-19 spread remain essential.

## 4. Discussion and lessons learned

While it is acknowledged that a short 30-s contact of saline with the SARS-CoV-2 virus does not exhibit direct virucidal activity (131), this narrative review on SI revealed that nasal SI performed repeatedly for 1 day may lead to a significant reduction in nasopharyngeal virus load, while a repeated application for 7–10 days leads to faster viral clearance. Overall, patients with positive swab tests experienced substantial viral load reduction, became PCR-negative faster, and had milder symptoms than untreated patients. Hence, repeated application of SI can be recommended for fastening viral clearance, symptom relief, and prophylaxis against respiratory infection, as has been increasingly proposed in (self-care) treatment or hygiene prevention guides for COVID-19 (9–13, 15, 59, 70, 79, 132–134).

Albeit found to significantly relieve symptoms in the study (53), and in line with pathophysiology (infection starting in the ciliated nasopharynx), short daily hypertonic saline gargling in COVID-19 patients did not fasten the SAR-CoV-2 nasopharyngeal clearance. In view of the low rate of live virus in gargle samples so far assessed in one study (76), larger-scale, well-validated studies are desirable to appreciate its potential role in reducing salivary loads and transmissibility in patients with COVID-19.

In the studies reviewed, both isotonic and hypertonic SI (1.5–3%) were used and found to be safe and effective for nose and throat irrigation in COVID-19, when administered with mildly pressurized flasks or devices, using protocols similar to those established in other respiratory diseases (5–13). Regular daily application of SI volumes, twice daily up to every 3 h rather than once daily, may enhance outcomes, as was observed in COVID-19 with comorbidity (80) or pneumonia (56).

Our evaluation of the mechanisms of saline and clinical studies suggests that saline may act more than placebo, similarly as previously proposed in other respiratory conditions (135, 136). This action may deserve attention in studies of nasal steroids, using saline drops or spray as comparator: A recent Cochrane analysis on the prevention of olfactory dysfunction was unable to conclude their efficacy (137). Whether combinations of saline with anti-inflammatory agents or corticosteroids provide extra-benefits over saline alone needs further study.

### 4.1. Additives

Overall, our data did not indicate a rationale for (antiseptic) additives to oral or nasal saline for daily oronasal hygiene (no significant intergroup differences) (46, 48–50, 53, 60, 61, 80). Possibly, the effect of additives may diminish fast due to salivary clearance (138). Many substances have been proposed for the development of nasal sprays or mouth rinses in COVID-19 (139). Bias through direct interaction with the PCR test by the additive should be excluded (see Supplementary Appendix A, p. 05, point 4). This is not a problem with saline which, in contrast, is used as a common PCR test sampling fluid.

### 4.2. Controversies and risks

Contrary to concerns that SI may cause harm through postnasal drip or contamination from nose to throat, respiratory tract, or olfactory bulb through viral movement or micro-aspiration (140–143), this review does not find evidence of such a risk. Moreover, this review did not find evidence for an increased hazard to contaminate or transmit SARS-CoV-2 through the use of nebulized saline for COVID-19, in the context of healthcare or household settings. The findings that viable virus is absent in (exhaled) bioaerosol during saline nebulization (75), and that transmission was not a problem during the clinical experiences with inhaled saline (121–124), support the guidance of authorities that encourage nebulization to be used or continued during COVID-19 (16). They also make nebulization of saline an acceptable alternative in (older) patients unable to perform SI. In Germany, pneumologists have acknowledged the effects of SI on bioaerosol since the start of the pandemic (132) and have encouraged patients to nebulize saline solution against COVID-19 (144). So, the use of SI or nebulization in the hospital or at home should not be discouraged, and this synthesis may help to inform authorities to adapt and/or adopt this hygienic measure.

Although data on SI for COVID-19 are far from complete, the results of this review show that there is solid translational evidence that for COVID-19, SI is unlikely to harm and likely to help. It is, therefore, reasonable, indeed ethically necessary, for authors of published and online news or recommendations on saline to amend erroneous claims suggesting harm for its use in COVID-19, for example (13, 140–143). The WHO has already taken this step by deleting SI from the Mythbusters webpage (14).

### 4.3. Limitations and developments needed

Most studies were small and of substantial heterogeneity, so bias cannot be excluded. However, given the overall robust *in vitro* and *in vivo* effects of nasal and oral SI, and the emerging evidence from observational and randomized studies (irrespective of saline being the object or reference/placebo), both health authority support and institutional funding are needed to design and perform large, randomized trials to formally test SI when implemented as an adjunctive preventive and treatment modality for COVID-19.

Yet, as SI use appears to be safe and is already practiced for common colds and viral upper respiratory infections (5–8), current evidence suggests that SI can be recommended as a public health measure along with other routine hygiene measures, albeit with strict attention to clean water and washing of SI materials. This is corroborated by experience with SI in COVID-19 in a hospital in China (59) and by a recent consensus of Chinese experts in otorhinolaryngology (15).

### 4.4. Practical recommendations

As an efficacious measure particularly if early started in COVID-19, SI can be effectively implemented at every age in those with positive SARS-CoV-2 test. We recommend its regular use as needed, up to 2–3 days after resolving symptoms, with a minimum of 10 days of repetitive saline nasal irrigation and gargling and repeated up to every 3 h in case of moderate COVID-19/pneumonia. Ideally, nasal irrigation and gargling are combined, as both nose and throat can be affected, and are started as soon as common symptoms develop and/or COVID-19 positivity is diagnosed. Larger well-standardized, randomized research trials may help to determine optimal volume, pressure, and salinity. Based on our analysis and the broader Chinese hands-on experience (15), any concentration between 0.9 and 3.0% appears to be suitable, while large, repeated volumes are preferred. For prophylaxis, several protocols have been suggested for use as part of the post-exposure or preventive protocol (15, 59, 70, 79), which can be all-inclusive (washing hands/face and saline eye rinse, nasal irrigation, and gargling), thereby aiming at covering all sites of viral entry or attachment (79). For video instructions how to make or apply SI at no cost, three links to video instructions are found under Supplementary Table S4.

## 5. Conclusion

Overall, substantial evidence was found *in vitro* and *in vivo* in support of SI use in COVID-19, also emerging from observational and randomized studies irrespectively of saline being the main intervention or reference/placebo in the study. As an international network of investigators with substantial clinical and research experience in saline use for several respiratory diseases and SARS-CoV-2 infections, we, therefore, propose to include rinsing the nose and throat with saline as an early intervention to prevent, and relieve symptoms of, SARS-CoV-2 infection: It is easy-to-use, inexpensive, safe, and feasible at every age. Considering the current high SARS-CoV-2 mutation rate and COVID-19 waves less responsive to population-based immunization, the recommendation to use nasal and oral saline irrigation is adjunctive to current biologicals and therapeutics, as well as to protective interventions, such as mask wear, social distancing, and hand hygiene. We also advocate for health authorities to dedicate further research funding to further clarify the preventive and therapeutic role of saline irrigation for SARS-CoV-2 infections at a global level.

## Author contributions

All authors of this Pro Bono Initiative, started by informal contacts per e-mail and Zoom, had pharmacological and/or clinical experience with saline irrigation in COVID-19 or related respiratory conditions. All the authors contributed by their knowledge, specific (pre)clinical research, or experiences (see reference list). Manuscript development occurred through the submission of work or description and discussion of mechanisms by e-mail and by Zoom meetings inviting all network members and coordinated by SH, SP, and DR. For completeness of the review, continuous searches for additional data were performed and new data were circulated by SH, while interpretations and problem-solving, development of lessons learned, and recommendations occurred through e-mail and discussion at the regular Zoom meetings. Integration of changes in the manuscript and revisions thereof were coordinated by SH, while regular feedback by e-mail to the Network and recirculation of the manuscript. All authors contributed to the development of consolidated knowledge, conception, and/or interpretation and helped in critical revision and resolving questions at each circulation until final consensus for submission. All authors listed have made a substantial, direct, and intellectual contribution to the work and approved it for publication.
