# Supplementary material for: Saline nasal irrigation and gargling in COVID-19: a multidisciplinary review of effects on viral load, mucosal dynamics, and patient outcomes
Source: Front Public Health. 2023 Jun 16;11:1161881. doi: 10.3389/fpubh.2023.1161881 (PMC10312243; doi:10.3389/fpubh.2023.1161881)
Supplement: Supplementary file 1 [file Data_Sheet_1.docx]

**Supplementary Appendix. Table of contents.**

1. Appendix A. Methods

A.1. Clinical studies with saline rinse ............................................................................. p.02

Search strategy ................................................................................................. p.02

Eligibility criteria................................................................................................ p.03

Table S1 (Study selection criteria, inclusion, exclusion) .................................... p.03

Data collection .................................................................................................. p.05

Number of studies eligible for tabulation, synthesis and review...................... p.05

Study stratification and analysis of outcomes .................................................. p.07

Data items tabulated & synthesis. ...................................................................... p.07

Study grading .............................................. ..................................................... p.08

Bias assessment .............................................. ................................................. p.09

Benefit-risk balance assessment, lessons learnt, recommendations................ p.09

A.2. Mechanisms of action ............................................................................................ p.10

Table S2. Study selection (inclusion, exclusion)................................................. p.10

2. Appendix B. Effect on viral shedding

Table S3. Clinical studies of saline rinse on viral shedding............................................. p.11

References of studies tabulated in Table S3 ................................................................. p.15

References of studies with saline (not listed) .............................................................. p.15

References to Legend of Table S3 ................................................................................. p.15

3. Appendix C. Clinical outcomes

Table S4. Clinical studies with saline irrigation (SI) or saline nasal spray (smaller volumes)

in COVID-19 by disease severity and special target groups .......................................... p.17

Video Instructions of SI, as used in some studies ......................................................... p.27

References of studies tabulated in Table S4 ................................................................. p.27

4. Appendix D. Hospitalization & ICU risks

Table S5. Rates of hospitalization and risks (Intensive Care Uptake, Pneumonia severity scores, Ventilation, Mortality) ................................................................................. p.30

Appendix A. Methods

Descriptive review, generated with systematic search strategies, (1) on the role of oronasal saline use in COVID-19 or SARS-CoV-2 infection, based on literature publishing on clinical outcomes with saline irrigation, nasal spray, gargling and/or nebulization in COVID-19 or SARS-CoV-2 infection, (2) as well on clinically relevant mechanisms how these outcomes may be achieved by saline. Nebulization was added in the search strategy to allow assessment of the harm-benefit balance of this procedure, often discouraged in COVID-19.^[[1]](#footnote-1)^

Particularly mechanisms relevant to the initial phase of SARS-CoV-2 infection were considered, at which stage saline irrigation or nebulization is likely to be initiated, in analogy to its (often traditional) use in common cold and respiratory disease.^[[2]](#footnote-2)^,^[[3]](#footnote-3),^^[[4]](#footnote-4),^^[[5]](#footnote-5),^^[[6]](#footnote-6),^^[[7]](#footnote-7),^^[[8]](#footnote-8)^ The project was started pro bono November 2021, and the Network was progressively built up through establishing contact between clinicians and researchers that published on the use or role of saline in COVID-19. All saline network participants thus had experience with saline in some respects.

Following iterative review of the identified studies, each topic and/or text sections by at least 2 reviewers through regular zoom meetings (January 2022-January 2023), new data were searched for, collected, and shared, and the findings were synthesized, reshared, and reviewed by all authors for consensus and recommendations, until final agreement on the text January 2023.

For the search strategy of clinical studies/reports, see A.1. and Table S1), summarized in Figure 1 of the main paper. For the mechanisms of actions, the search strategy, is explained under A.2., summarized in Table S2. The results have been schematically consolidated in Figures 1-4 of the main paper.

**A.1. Clinical studies with saline rinse**

*Search strategy*. Information related to saline and of relevance to COVID-19 or SARS-CoV-2 infection was broadly retrieved electronically using internet searches from 1 March 2020 to 27 October 2022 of the following databases: PubMed, Research Gate, ClinicalTrials.gov, and Google in general. The literature on saline itself is abundant, as saline is used in experimental in vitro and in vivo research, in formulations and chemical analysis, synthesis, such as a common eluent, as well as intravenously for medical purposes or drug administration, and commonly as placebo, even in vaccine studies. Hence, total sources retrieved during the rolling review (often iteratively rescreened at a later stage) were not counted and down-filtered by combinations of MESH terms with COVID-19 and/or SARS-CoV-2.

MESH terms for primary systematic searches included: gargling and/or (mouth) rinse/wash, irrigation and/or lavage (SI), nebulization and/or nebulizing *and* SARS-CoV-2, COVID-19, *and* saline; these searches were regularly expanded with terms such as nasal spray, or nasal drops, of which only those sources that were clearly mentioning the use of saline were retained (often used as placebo or reference treatment). Broader search terms on the internet, combined with the main terms, also included viral load, clinical study or trial, salt, sodium chloride, NaCl, isotonic, hypertonic, and respiratory infection or disease, if applicable. Retrieval of additional references based on review of published reviews or opinions was allowed as well. Lateral targeted searches for references were performed, allowing the inclusion of any word of relevance (e.g. *Naglaeria or N. fowleri*, blood pressure, dangerous...) to document potential risks, adverse event, and myths of saline use (most relevant source(s) or a few examples retained).

*Eligibility criteria.* For being eligible, the sources had to study SAR-CoV-2 qPCR-positive patients (except if prophylaxis), and had to contain information allowing to evaluate (1) effect of saline (any type as defined per MESH terms) on viral load and clearance and (2) clinical outcomes, such as symptom resolution, recovery, relief or symptom severity (any type of rating), pneumonia severity score (any type of assessment) , hospitalization, admission to intensive care, or oxygen needs and/or mechanical ventilation, and mortality (percentage of patients).

All inclusion and exclusions criteria that were developed and used throughout the review exercise are listed in Table S1. The exclusion criteria grew, the more insights were gained during the assessments during the networking. Data on (human) coronavirus other than SARS-CoV(2), or studies with only part of patients tested by qPCR (so not asserting the reported outcomes apply to COVID-19 patients), were not retained. For prophylaxis, SARS-CoV data were allowed, in view of their genetically close relationship with SARS-CoV-2,^[[9]](#footnote-9)^ and prior experience identified in the literature (no data found on use in SARS-CoV patients).

For the sources on SI, saline spray use or drops, gargling or nebulization in COVID-19, we did not limit the selection of sources by language or study type and included also reports of hands-on experience and compassionate use. Single case reports were not retained.

| **Table S1. Study selection criteria, Clinical Outcomes Saline Irrigation/nasal sprays in COVID-19/SARS-CoV-2 infection:**   - PubMed and broad general Internet searches, combining with COVID-19: saline +/- irrigation, gargling, nasal, spray or drops, nebulization/nebulizing, inhalation, mouthwash/rinse [started by one author 1.6.2020, repeated more systematic at least bi-monthly: 1.01.2022 - 27.10.2022]. Broader search terms, combined with the main terms, also included viral load, clinical study or trial, salt, sodium chloride, NaCl, isotonic, hypertonic, (saline) placebo, and respiratory infection or disease, if applicable. - Primary searches: see Figure 1 - Cochrane searches - WHO and Clin.gov data bases (search for completed and ongoing studies, December 2021)   **Eligible studies**   - All types of reports, also in other language, if English abstract available or translation allowed reliable retrieval of data for evaluation |
| --- |
| **Inclusion:**  Clinical studies:   - Patients with SARS-CoV-2 infection, identified by qPCR ( except if prophylaxis) - Any source reporting on qPCR-positivity or clearance in individuals - Any source reporting on clinical outcomes |
| Intervention   - Saline: saline irrigation, lavage, nasal spray use, gargling, mouth rinse, inhalation or nebulization - Acute treatment or prophylaxis |
| Comparison:   - Absence of saline, saline irrigation - No intervention - Standard of care (can be antipyretic, an antiviral or other treatment used for COVID-19) - Nasal corticoids - Other nebulization treatments, unless excluded (see exclusion) |
| Types of studies:   - Randomised controlled trials (RCTs) - Non‐randomised (matched) controlled studies (nonRCTs) - Prospective and retrospective studies - Hand-on experience - Compassionate use - Pilot (pre- , post) studies - Cross‐sectional studies |
| **Exclusion**:  Were excluded from the Tables:   - All sources making clinical efficacy claims based on in vitro assessment, animal data, ideas, concepts, perspectives, or review material, such as based on trials with saline in respiratory infections other than SARS-CoV(2), chronic diseases, or proposing additions to saline; these were screened, and only eligible studies were retained. [Reviews on SI in COVID-19 were screened for eventual trials or reports of use in SARS-CoV-2 infected patients] - Sources related to SI use for diagnostic detection, sample collection or broncho-alveolar lavage - Observations made during other procedures such as surgery or other diagnoses in COVID‑19 patients - Sources using saline for evaluation of development of devices, - Sources on studies only reported in lay-press (these are only mentioned in the text as such if relevant, but not tabulated) - Single case reports or very small randomized clinical comparator studies assessing symptomatic outcome based on less than n< 10 patients per group, in view of the substantial heterogeneity in symptom severity developed during COVID-19 |
| Were also deleted upon screening :   1. All studies not relating to oral, nasal, oronasal administration or nebulization/inhalation of saline (e.g. systemic use, if given intravenously (iv) or if used as placebo for vaccine studies) and/or if used as carrier of a drug in development. 2. All ‘treatment’ studies with electrolysed saline (ES), if also using systemic ES (SES), by intravenous injection in a part of the patients: these were deleted as the impact of iv administration on the results is not clear; one trial on prophylaxis was maintained (because no iv use and very low chlorine and the protocol mentioning ‘saline’ as intervention) 3. Comparisons (mainly of corticosteroids) with saline dry powder or only dry metered dose inhalers of a saline placebo; if using saline spray or drops (small volumes allowing minimal hydration) the study was maintained. 4. Comparisons of nasal drops or sprays or gargles containing nitric oxide, carrageenan, herbal antivirals or other active compounds, such as surfactant, claiming benefits of these additions, were not retained for following reasons:   (1) often or possibly containing saline as part of their own formulation (not always disclosed)  (2) if saline placebo-controlled, uncertainty whether the same saline composition was present in the placebo  (3) the articles did not disclose validation of the reverse-transcription (quantitative) Polymerase Chain Reaction (rt-(q)PCR test), while no care was taken to the timing of swabbing/sampling for quantitative viral load PCR-testing. This needs to be standardised as many of the substances may interfere with the PCR-test (suppress reverse transcription or amplification), so possibly leading to false PCR-negative results or to altered Ct-values (lower titers) upon quantitative assessment of viral shedding. For instance, a study of a complex essential oil (saline) nasal mixture showed that PCR-positivity falsely dropped from 15% to below 0.5% due to interference of nasally swabbed compounds impairing the PCR-test, making the study investigators switch to the assessment of a different endpoint (positive serum immunoglobulin G specific to the spike protein of SARS-CoV-2).^[[10]](#footnote-10)^ The PCR-tests thus need thorough validation, as has been previously described,^[[11]](#footnote-11)^ at low concentrations of substances added and expected to be collected in the (oro)nasal specimen. Loss of positivity in qPCR testing or a decrease in Ct-values can for instance be expected - based on prior independent research - for nitric oxide,^[[12]](#footnote-12)^ and carrageenan.^[[13]](#footnote-13),^^[[14]](#footnote-14)^ In contrast, saline has no major impact on the performance of gargling and (oropharyngeal–)nasopharyngeal swabs for SARS-CoV-2 RT-PCR testing.^[[15]](#footnote-15)^ The role of standardization of the sampling time for PCR-tests has been recently illustrated, for PVI gargling, by determination of the salivary SARS-CoV-2 RNA Ct-values and viral titers up to 120 min.^[[16]](#footnote-16)^   1. Protocols of non-performed or no longer pursued studies (e.g. NCT04341688, Khan 2020^23^,ELVIS COVID-19 Study^24^) 2. Post-Covid (Long COVID) studies, focusing at study entry on persisting symptoms beyond 2 to 4 weeks from the onset of COVID-19 symptoms (e.g. NCT04789499 (Gupta 2022)) |

*Data collection.* Main searches were regularly performed by one person (at least monthly), but all members of the network regularly provided also articles based on their own lateral searches. Pdfs of studies retained for evaluation and synthesis were shared on a Google Drive, accessible by direct link, and discussed during zoom sessions, and subsequently tabulated and revised in detail by at least one other reviewer.

*Number of studies eligible for tabulation, synthesis and review.* In total, 33 clinical studies of saline rinse (gargling, irrigation or sprays/drops) or nebulization in COVID-19 were identified spanning various study designs, disease severity, irrigants or nasal spray/drop comparators, and outcomes, their heterogenous nature however rendering the pooling of the data or meta-analysis difficult. To allow easy insight in the variety of designs, an algorithm is presented by type of study result in Figures 3 and 4 in the main paper.

- Fifteen studies were identified allowing to evaluate the effect of saline on viral load or clearance (tabulated in Table S3, p.11): four studied gargling; ten saline irrigation (n=8) or nasal spray (n=2). See Figure 3: the studies were split out by type of load (salivary, nasopharyngeal or mid-turbinate) and by randomised controlled trials (RCTs) and non-randomised controlled trials/studies (nonRCTs)]; one study rather assessed the clearance of the combined use of an antiviral plus saline irrigation versus irrigation alone.^^[[17]](#footnote-17)^^
- Twenty two clinical studies or reports in SARS-CoV-2 infected patients reported on patient-oriented benefits, using (oro)nasal SI (n=9), nebulization (n=4), nasal saline sprays or drops (sometimes as placebo or reference) (n=8) or gargling (n=1), the studies tabulated by disease severity in Supplement Table S4, Section B, p.19 : to note, eight of these studies also reported on viral load or clearance in the patients (so also covered in Table S3). For overview of the type of studies by use and disease severity, see Figure 4.
- One of the above clinical studies also reported on household transmission as secondary outcome. Including the latter study, a total of five studies were listed in Table S4, section A, p.17relating to prophylaxis (one of which with SARS-CoV).
- An extra study, not listed in Tables S3 and S4, assessed

the effect of isotonic saline nebulization on viral load/risk for dissemination in bio-aerosol in COVID-19 patients and is discussed in section A4.^^[[18]](#footnote-18)^^

This number of studies or reports identified by our broad iterative search strategy, is substantially higher than found in a recent systematic review from Gandi et al.,^[[19]](#footnote-19)^ searching for studies related to nasal irrigation, viral illnesses, and types of nasal rinse (including saline, corticosteroid, and polividone-iodine) in ClinicalTrials.gov, Cochrane, Web of Science, Embase, and MEDLINE database up to April 2022 (also retaining non-COVID + non-SARS-CoV(2) sources). They analysed twelve studies (n=10 RCTs), eight examining COVID-19. Of the RCTs, six used saline, three polyvidone-iodine, and 1 one an intranasal corticosteroid. The details/results of this review have so far not been published (only congress abstract available) and are therefore not referred to or discussed in our article.

Cochrane data base search only resulted in 2 sources: one Cochrane report dating from 2020 targeting various (antiseptic) irrigation formula, yet finding no studies completed while various (RECOVERY) outcomes were driven by the (adverse) profile of antiseptics or other additions, ^[[20]](#footnote-20)^ the other report dating from 2022 on interventions for the prevention of persistent post-COVID-19 olfactory dysfunction (only one source relating to saline).^[[21]](#footnote-21)^ All relevant sources from these reports, related to saline, were also identified through our search strategy. At last, although many reviews and meta-analysis have been published on mouth rinses with antiseptics, these are not discussed in our review, as these generally do not include saline and rather focus on viral shedding immediately to several hours after single use for dentistry practice purposes.

Clin.gov and WHO databases were screened January 2022 for relevant clinical studies, also with the objective to find out whether saline was subject of a public or developmental programme to study saline as hygiene measure for COVID-19. This seemed not to be the case. The number of potentially useful studies retrieved included seven hits in the Clin.Gov protocol database (2 studies not recruiting, one completed and retrieved on PubMed) and 16 hits in the WHO clinical database: three were still recruiting, 13 not recruiting (search results available on request).

*Study stratification and analysis of outcomes.* As the rinse effect is the major presumed mechanism, the clinical studies were further stratified for tabulation en evaluation as follows:

| All relevant information, even if of poor reporting quality, was stratified in tables by effects on:  (1) SARS-CoV-2 viral load or shedding (change in titers/qPCR Ct-values) or clearance (time at which one becomes qPCR-negative) in saliva or nasopharynx  (2) viral load in exhaled bioaerosol  (3) viral load related: prophylaxis (protection Health Care Workers) and secondary household transmission  (4) Patient-oriented clinical outcomes:  Symptom relief/resolution, as COVID-19 patients suffer typical common cold symptoms relieved by saline  Relief/resolution of taste/smell disturbance(s), typical of SARS-CoV-2 infection though not always present  Risks: pneumonia severity or worsening, rate of hospitalization, Intensive Care Unit (ICU) admission, need of (invasive) oxygen, mechanical ventilation and/or mortality. |
| --- |

*Data items tabulated & synthesis.* Were tabulated: type of patient (outpatient or hospitalised, severity disease, formulation and treatments used, trial design and patient numbers by study group. If the registry number of the protocol was mentioned or could be identified, this reference was added; (otherwise the approving committee or procedure). Clinical outcomes were reported as stated in the source. No interpretation or weighing of data was performed. If concerns about bias, missing statistics, robustness of the data or incorrectly claimed benefits, this was mentioned under concerns.

The effects of saline on viral shedding/clearance are tabulated in Table S3 (Supplementary Appendix Part B), further stratified by rinse type/volume and regimen. The patient-oriented outcomes have been listed in Table S4 (Supplement Appendix Part C) by disease severity or special risk groups or symptoms focused on. Table S5 (Supplementary Appendix Part D) consolidates only relevant risk outcomes (hospitalization, Intensive Care Unit (ICU) admission, need of (invasive) oxygen, mechanical ventilation and/or mortality), from the studies tabulated in Tables S3 and S4 whenever applicable. Compilation of data in the narrative review was performed by qualitative analysis of the tabulated data.

*Study grading.* Study grading was attempted by 3 reviewers following the algorithm according to Ebel et al. 2004.^[[22]](#footnote-22)^ Yet, the grading approach appeared not to be applicable, as we found no large, randomised studies assessing SI, of the magnitudes in patient numbers as currently applied in drug developments for COVID-19, while the randomised comparator studies usually used saline rather as a reference or placebo. To date, only protocols for such large trials have been published.^[[23]](#footnote-23),^^[[24]](#footnote-24)^ We suspect (and as experienced by investigators of the Network) that funding challenges, the regulatory and ethics challenges associated with human subjects’ research in the COVID environment, the WHO mythbuster status of saline irrigation, or communications of SI designated as fringe or unscientific therapy, all limited conduct of such trials. Moreover, as pointed out, the studies were of substantial heterogeneity (1) regarding study design, outcome assessment and hygiene practised, which is not surprising in view of the various low/high volume, low/high pressure formulations and devices currently available and used for nasal hygiene in upper respiratory tract infections^2^, and (2) with regard to reporting quality, likely because the local nature of initiated studies, not being part of a coordinated research and development programme. Hence, most of our findings and recommendations rely on the evaluation of smaller empirical or local investigator-initiated studies or hand-on experience in Polymerase Chain Reaction (PCR)-positive patients enrolled after a positive PCR- test, assessing SI, nasal spray use, gargling or nebulization. Studies using electrolysed saline, or 0.9% saline as placebo are mentioned, if informative to the outcomes.

*Bias assessment*. If a bias in outcomes was suspected, this was mentioned under ‘Comments’ in the Tables S3 to S5. Because patients were enrolled in the first place following a SARS-CoV-2 PCR-positive test, bias by sequence generation, absence of blinding of participants and personnel, and attrition bias by allocation concealment, were considered unlikely to favour saline use, this even more so in studies where saline was used as reference or placebo. Bias in interpretation of the outcomes was further avoided by listing and evaluating the studies by disease severity and/or targeted outcome. Notwithstanding, unknown risks for bias may exist in non-randomised studies, comparing to cohorts or matched controls, and open explorative cohort studies. Yet, even if such biases would be present, it is unlikely that these affected the results and risk–benefit ratio, as reported under Lessons Learnt,

*Benefit-risk balance assessment, lessons learnt, recommendations.* Clinical benefits, risks, lessons-learnt and recommendations were formulated through consensus with the written statements, after evaluation and discussion of the benefit – harm balance of each point, based on the consolidated findings from the tabulated data and additional laterally retrieved references if needed. Thereby an approach was taken for the formulation in line with those used in the literature for graded studies.^[[25]](#footnote-25),^^[[26]](#footnote-26)^ In the case of more benefits found than harm, these were positively formulated, provided the findings were consistent throughout randomised and non-randomised studies. They were phrased conditional (‘data suggest...’; ‘to be confirmed’), in the case an overall benefit was suggested, but (1) the effects observed across studies were not observed, but no consistently statistically significant, (2) the parameter (hospitalization....) was not a primary parameter documented in large randomised studies, and/or patient sample sizes of the studies were too small to ascertain the outcome, so requiring larger studies to confirm the effect, so demanding further study. No recommendations on iso- versus hypertonic saline were formulated: both were used throughout the studies, with no clear-cut benefit-harm balance deducible towards one or the other formulation. Similarly, we made no recommendations on the benefit-harm of additives – found to be a complex study by itself, as a preliminary analysis proved to be for calcium chloride and hyaluronic acid (preliminary findings available on request).

**A.2. Mechanisms of action:**

For pharmacological or (pre)clinical mechanisms, judged clinically relevant in initial mucosal stage of SARS-CoV-2 infection, sources were retrieved through progressive literature searches on the topic investigated and information was retained, if relevant to saline and NaCl (sodium chloride) *AND* to the (patho)physiology and transmission of COVID-19, and further also if previously documented as mechanism of saline in other respiratory disease, if relevant to the (patho)physiology of COVID-19.

While the literature on saline and COVID-19 is extensive, systematic searches on target words often did not result in relevant information, while insights grew during the reviewing process, also due to active broad literature reading and screening on COVID-19 (the effect NaCl often passing unnoticed, or not the topic of the articles). As NaCl is ubiquitous in the human body, playing a role in many processes, only the most representative source(s) to the topics covered in our review were retained. As the physiological effects of NaCl on the immune system are multiple and complex, only literature on the effects on neutrophils en NETS (neutrophil extracellular traps) were retained in view of their involvement in the deterioration in COVID-19. Direct effects of NaCl on oxygen sensing, platelet aggregation and the complement system were not reviewed. The mechanisms identified are schematically presented in Figure 2. Haemodynamics or more systemic processes were not covered.

| **Table S2. Study selection Mechanisms of saline/ NaCl in COVID-19/SARS-CoV-2 infection:**  PubMed and broad general Internet searches, combining saline/NaCl with COVID-19 and/or respiratory disease or conditions [1.6.2020 - 27.10.2022].  Main topics (of potential relevance to mucosal dynamics): |
| --- |
| Inclusion: Local mechanisms relevant to initial infection (yet, not limitative):   - Cell models infected with SARS-CoV-2 - In vitro assessments of relevant proteases (3Clpro, Mpro, furin) - Studies in COVID-19 or SARS-CoV-2 infection documenting the role of micro-aspiration of virus/secretions from nose to deeper airway/lungs - [Representative source(s)] Mechanism relevant to NaCl, saline and COVID-19 relevant to :   - Bio-aerosol formation   - Viral replication (membrane potential; not retained : effects on liquid-liquid phase separation)   - Hydration   - Mucociliary clearance, mucus and cough clearance (+ mucus+hypoxia) – (Micro)-aspiration   - ENaC channel (inhibition) – Na helper channels   - Neutrophils/Neutrophil Extracellular Traps (NETS)   - Hypochlorous (HOCl) production |
| Excluded (not covere):   - Modulating effects of NaCl on other (patho)physiological mechanisms and more systemic effects, even if possibly relevant, such as:   - Mechanisms whereby behaviour of cells studied is often differing and modulated by the conditions used in the in vitro model:     - Immune cells other than neutrophils, such as macrophages, B-cell and T cell differentiation     - Complement system     - Platelet aggregation     - [not limitative, as there are many more processes in which NaCl is involved] |

Appendix B. Effect on viral shedding

**Table S3. Clinical studies of saline rinse on viral shedding.**

For study retrieval, see supplement Part A. Studies are listed by (A) single and repeated applications: (B) repeated gargling alone, and (C) repeated oronasal irrigation or nasal spray: C.1. Asymptomatic subjects; C.2. Symptomatic mild COVID-19 (irrigation); C.3. Symptomatic mild COVID-19 (nasal spray); C.4. Moderate to severe COVID-19 presenting at hospital. (E): Electrolysed saline [Not discussed in Review]

| ***Saline use***  ***Route***  ***IS=0.9% NaCl***  ***HS>3% NaCl*** | ***Study design***  ***Type of sampling***  ***Type and number (N) of PCR positive (PCR+) patients/cohabitants*** | ***Outcomes*** |
| --- | --- | --- |
| **A. Single(-day) gargles or nasal rinses** | | |
| A.1. Single gargles | | |
| Gargle  IS control  2 x 10 mL for 30 sec  *Natto et al. 2022*  NCT04941131 | Randomised controlled 4-arm study:  chlorhexidine gluconate (CHX) mouth rinse; lozenges, PVI-I, 0.9% saline control.  Salivary load at baseline and immediately after rinse (<5 min) | Decrease of salivary viral load in all 4 groups combined (*P*-value for E genes =0.027, and for S genes =0.006).   - PVP-I and CHL, but not saline (starting at higher baseline Ct) showed significant reduction in Ct values versus baseline (*P<*0.05) - No significant intergroup differences (P >0.05) |
| Gargle  IS control 0.9%  20 mL for 30 sec  *Sevinç Gül et al. 2022* | Randomised controlled trial: effect on salivary viral load comparing:   - HClO (n=20) - PVP-I (n=21) - N=20 saline gargle (n=20)   Salivary load in saline sample at baseline and immediately after rinse  [n=14 saline gargles PCR- at start]] | - HClO and PVP-I: significant negative PCR-test results vs baseline (p < 0.05) - No significant intergroup differences (p > 0.05) |
| Gargle  IS  2 x 7.5 mL for 30 sec  *Chaudhary et al*. *2021*  NCT04603794 | Tripple-blind randomized study comparing IS, 1% H_2_O_2_, 0.12% chlorhexidine and 0.5% PVD-I;  Saliva 15 and 45 min after gargling:   - N=40 (10) symptomatic (N=10/group) | Median (mean) reduction of salivary viral load:   - IS= antiseptics, significant reduction by all 4 mouthrinses (*P*<0.05) - At 15 min: 61% - 89% (25%-74%); at 45 min: 70% -97% (30%-43%); values with IS: 89%-90%, resp. - Extent correlated with initial viral load; if baseline viral load < 10^4^/mL copies (n = 6): 100% reduction at 15 and 45 min |
| A.2. One day (6-16 hrs nasal SI | | |
| Nasopharyngeal SI  IS – 10 mL bottles  Every 4 hrs x for 20–30 sec for 16 hours, 10 mL in each nostril  *Pantazopoulos et al.2022*  NCT05525832 | Randomised controlled prospective study  NPS PCR test, at baseline and 8 hours after last wash (24 hrs later in controls):   - *N=50* (N=24 saline, N=26 controls – controls were older on average) - Hospitalized patients with COVID-19 pneumonia - Testing before and after by the same doctor, N gene Ct values | Mean change in Ct-values versus baseline:   - IS: 8.9% increase (*P*=0.007) (=reduction of load) - Controls: 9.7% decrease (*P*=0.01)(=increase) - Difference between both groups (*P=*0.005)   At follow-up (2 weeks after hospital discharge):  15/24 SI versus 6/26 controls were PCR- (*P* = 0.02)  For clinical follow up : see Suppl. Table S4 (less escalation in respiratory support, ICU and mortality, although not significant difference for these small patient groups) |
| Nasopharyngeal SI  HS (5%) – 25 mL bottle,  3 x for 20–30 sec within 6 hrs  *Vantarakis et al. 2021*  Ethic Approval Bord, No. 11055, 23/04/2021 | Open prospective case-control study  NPS PCR test:   - N=25 (N=20 saline; N=5 controls) - Mild to moderate disease, hospitalised, receiving oxygen support via Venturi Mask - Testing before and after by the same doctor | Median (mean) change (reduction) of NP viral load:  - HS: 23.6% (17.3%) (broad range of Ct-values)  - Controls: Ct values unchanged  (patient samples small, while large variation in data; *P*>0.05)  [Comment from co-author Dr. Poulas: the range of values for viral load reduction obtained with hypertonic saline was much larger than found in the study with isotonic saline by Pantazopoulos et al . Reason unknow. Some had only low reductions, possibly because some patients may have performed the rinse procedure less rigorously because of lower tolerability of hypertonic saline?] |
| **B. Repeated gargling alone – (a)symptomatic subjects mild COVID-19** | | |
| Gargling  HS  20 mL/15 sec  3x daily  *Chalageri et al. 2022*  CTR India /2020/09/027687 | Randomised comparative study (blinded by sealed envelopes). Comparators:   - Inhalation with electronic, automated steam inhalers, 3–5 min - Gargling 36 ml PVI 0.5%/30 sec - Controls: antipyretic, antibiotics, zinc supplements, and Vitamin C (SOC)   Nasal and oropharyngeal swabs:   - N=80, 65 (81.3%) symptomatic   (N=20/group) | Clearance (2 nasal + oropharyngeal PCR- consecutive samples): no significant differences despite significant symptom recovery with saline gargling only (see Suppl. Table S4)  Median time till PCR-: HS gargling once 15 sec = Steam inhalation = Controls receiving SOC (median: 9 days)  = PVI once 30 sec (median: 6 days) (*P*=0.8)  [To note: potential bias in favour of PVI: larger volume and twice as long gargling instructed with PVI than for gargling with saline] |
| **C.1. Repeated nasal rinses/irrigation – asymptomatic subjects** | | |
| Nasal nebulization with nasal douche device Rinowash^©^:  HS (3%)^+^  twice daily for 7 days  [+^+^xylitol,hyaluronate]  *Ciprandi et al. 2021* | Open controlled study in households  NPS PCR test after 7 days:   - N=172 asymptomatic patients/cohabitants:   N=72 using HS from day 7 to 14; N=63 using HS from day 14 to 21;  N= 37 controls | Clearance : % patients PCR- after 7 days:   - HS: 100% (PCR- on day 14, if rinsing from Day 7 to 14, on day 21 if rinsing from Day 15 to 21) - Controls: all PCR- by Day 28.   See Suppl. Table S4 for clinical recovery |
| **C.2. Repeated nasal rinses/irrigation (large volumes) – symptomatic subjects mild COVID-19** | | |
| Nasal SI  Lavonase^©^  IS 250 mL  once daily for 10-17 days  *Spinato et al. 2021*  Treviso and Belluno provinces ethic vote: 871/CESC | Non-randomised case-control study  NPS PCR test 10 days after diagnosis; if still positive, another test 7 days later:   - N=140 mild symptomatic patients (N=70 saline; N=70 historical controls matched for age, sex and baseline symptoms) - Treatment started day of test | Clearance : % patients PCR- at 10 days   - IS: N= 62/70 (91.1 %) - Controls: N= 2/70 (2.8%)   (*P < 0.0001*)  See Suppl. Table S4 for clinical recovery |
| Nasal SI  HS (McNeil buffered salt)  volume not reported  twice daily for 21 days  *Esther et al. 2022*  NCT04347538. | Open randomised controlled comparative study  Comparing nasal SI with HS or HS/J&J shampoo and no intervention  Mid-turbinate PCR self-swabbing:   - N=72 ambulant symptomatic (N=24/group) - Self tests repeated over time at home but obligatory *postponed for 4 hrs “after” irrigation* in the 2 irrigation groups | Evolution of mid-turbinate viral load (Ct):   - HS = HS/J&J shampoo = controls - Ct values quickly evolving to high Ct>30 (=low viral loads) – Inappropriate self-sampling & storage? - No data on % patients PCR-, yet number censored Ct>50 on day 7 is highest in HS group - No effect of topical detergent added to HS   [To note: study inconclusive for HS versus controls: no baseline values, low mid-turbinate self-swabbed viral loads (storage?), no acute rinse effect assessed as swab was obligatory delayed 4-hour after SI (also potential bias by circadian rhythm***] |
| Nasal nebulization with nasal douche device: Rhinowash^©^  HS (3%)^+^  twice daily for 7 days  [^++^xylitol, hyaluronate]    *Varricchio et al. 2021* | Open controlled study  NPS PCR test, repeated after 10 days:   - N= >152   N= 76 saline mild symptomatic  N = >76 asymptomatic cohabitants as controls   - Symptoms < 3 days prior to inclusion | Clearance : % patients PCR- at 10 days:   - HS use: 100% - Controls: none (all still PCR+) after 10 days   [To note: patients also received antibiotics (tobramycin/lincomycin)]  See Suppl. Table S4 for clinical recovery |
| **C.3. Repeated nasal drops/spray (smaller volumes) – symptomatic subjects mild COVID-19** | | |
| Nasal spray  IS  2 sprays per nostril daily, four times daily for 5 days  *Zarabande et al. 2021*  Stanford University  NCT04347954 | Triple-blind randomized placebo-controlled comparative study  Comparing nasal spray IS (placebo), 0.5% PVP-I and 2.0% PVP-I  NPS (Ct) at baseline, after 1 h and day 3:   - *N=45* symptomatic (N=15/group) | Reduction of NP viral load:  0.9% IS = 2.0% PVP-I > 0.5% PVP-I   - not significant 1 hr after first spray application versus baseline - significant for all 3 sprays after 3 days of application: - (no significant differences between treatments)   See Suppl Table S4. for clinical recovery |
| **C.4. Repeated nasal rinses/irrigation (large volumes) – symptomatic subjects with moderate to severe COVID-19 presenting at hospital** | | |
| Nasal SI  IS daily  *Cao et al. 2022* | Open controlled study  Nasal PCR test until 2 subsequent PCR- samples  Survival analysis   - N=68 SI - N=72 controls | Clearance : patients on daily nasal SI faster PCR- than controls (*P*<0.001) |
| Nasal SI  IS, 250 mL (Jal Neti)  increased frequency – dose finding (b.i.d. increasing to at least every 3 hours)  *Chatterjee et al. 2020*  CTRI/2020/08/027465 | Open prospective randomised dose-finding study  NPS PCR test:   - N=125 presenting at hospital with pneumonia without ARDS (N=62 saline, N=63 controls) further treated as outpatients | Clearance : % patients PCR- at 10 days:   - IS (overall): 48% - If rinsing with IS every 3 hrs: 70% - Controls: 25% (*P*<0.05) |
| Nasal SI  Alkaline HS  2.3%, pH 10  one puff each nostril  4x daily for 7 days **  *Yilmaz et al. 2021*  Scientific Research Projects Coordination Unit of Istanbul University-Cerrahpasa, Project number: 34932 | Randomised controlled study (clinic and laboratory staff blind to randomization status)  HS add-on to HCQ versus HCQ alone  NPS PCR test at baseline, day 3 and 7:   - N=60 hospitalized patients with no or mild lung involvement on CT scan but without dyspnoea/sO2 <95% (N=30/group) | Changes in median NP viral load:   - Decreased faster with saline: - Day 3: significantly higher reduction with HS than in control group (*P*=0.019) - Day 7: further decreased in both groups with median in HS group tending to be lower than in controls (*P*=0.067); reduction no longer statistically significant between groups   See Suppl. Table S4 for clinical recovery |
| Nasal SI  IS daily  *Zou et al. 2022*  ChiCTR2200056817 | Open controlled study, comparing molnupiravir with controls, both groups also recommended daily nasal SI  Nasal PCR test until 2 subsequent PCR- samples  Survival analysis   - N=77 (N=76) molnupiravir + SI - N=31 SI controls | Primary endpoint: median days until 2x PCR-:  9 days with combination, compared to 10 days with SI (*P=* 0.0092)  Clearance : patients on combination faster 2x PCR- than SI controls (*P<0.001*)   - Day 5: 18.4% versus 0%, resp. (*P*=0.0092) - Day 7: 40.8% versus 6.5%, resp. (*P*=0.0004)   No data on (1) baseline duration pre-existing symptoms (2) frequency and compliance of nasal SI |

Abbreviations: IS = isotonic saline, HS = hypertonic saline (3-5%); HCQ = hydroxychloroquine; SOC =standard of care; study design: N = number of qPCR-positive patients enrolled (N=numbers per group); NPS = nasopharyngeal swab specimen; PCR+ = positive for SARS-CoV-2; PCR- = negative for SARS-CoV-2. Results: Ct = Cycle threshold: Ct denotes how many PCR cycles are required before the SARS-CoV-2 viral RNA reached a detectable level; higher Ct values correspond to lower viral copy numbers (for reference, Ct values of 20 correspond to ~2.12 x 10^6^ viral copies per mL, while a Ct value of 40 is undetectable and is considered the lower limit of detection of the RT-PCR test for SARS-CoV-2); CT scan = Computed Tomography scan of lungs.

* Device with a containment chamber to collect the liquid returning from the nasal cavity

** Pure Lake Van water, the Earth's largest soda lake in Turkey, with high salt (23 g/L) and pH (about 10) levels

***Esther *et al.* 2022: lower mid-turbinate viral loads (Jamal et al. 2020, Pinninti et al. 2021), inaccurate self-swabbed or unobserved sampling (Abdollahi et al. 2020; Kinshella et al. 2022) and inappropriate storage conditions may have annihilated differences in viral titers (Agaoglu et al. 2022); circadian rhythm Ct values - highest loads at noon (McNaughton et al. 2021, Zhuang et al. 2022).

**References of studies tabulated in Table S3 (alphabetical):**

Cao J, Wen M, Shi Y, et al. How should designated COVID-19 hospitals in megacities implement a precise management strategy in response to Omicron? *Biosci Trends.* 2022; 16(3): 242-244. doi:10.5582/bst.2022.01261.

Chalageri VH, Bhushan S, Saraswathi S, et al*.* Impact of Steam Inhalation, Saline Gargling, and Povidone-Iodine Gargling on Clinical Outcome of COVID-19 Patients in Bengaluru, Karnataka: A Randomized Control Trial. *Indian J Community Med.* 2022; 47(2): 207-212. doi: 10.4103/ijcm.ijcm_804_21.

Chatterjee U, Chakraborty A, Naskar S, Saha B, Bandyapadhyay B, Shee S. Efficacy of normal saline nasal spray and gargle on SARS-CoV-2 for prevention of COVID-19 pneumonia. *Research Square.* Preprint 2021; PPR: PPR277214. doi:10.21203/rs.3.rs-153598/v1 https://www.researchsquare.com/article/rs-153598/v1

Chaudhary P, Melkonyan A, Meethil A, *et al.* Estimating salivary carriage of severe acute respiratory syndrome coronavirus 2 in nonsymptomatic people and efficacy of mouthrinse in reducing viral load: A randomized controlled trial. *J Am Dent Assoc.* 2021; 152(11): 903-8. doi: 10.1016/j.adaj.2021.05.021.

Ciprandi G, La Mantia I, Brunese FP, Varricchio A, Varricchio A. Hypertonic saline with xylitol and hyaluronate may shorten the viral shedding duration in asymptomatic COVID-19 positive subjects: a pilot study*. J Biol Regul Homeost Agents.* 2021; 35(3): 1151-4. doi: 10.23812/21-138-L https://pubmed.ncbi.nlm.nih.gov/34229425/

Esther CR Jr, Kimura KS, Mikami Y, et al. Pharmacokinetic-based failure of a detergent virucidal for severe acute respiratory syndrome-coronavirus-2 (SARS-CoV-2) nasal infections: A preclinical study and randomized controlled trial. *Int Forum Allergy Rhinol.* 2022: 10.1002/alr.22975. doi: 10.1002/alr.22975

Natto ZS, Bakhrebah MA, Afeef M, et al. The short-term effect of different chlorhexidine forms versus povidone iodine mouth rinse in minimizing the oral SARS-CoV-2 viral load: An open label randomized controlled clinical trial study. *Medicine (Baltimore).* 2022; 101(30): e28925. doi: 10.1097/MD.0000000000028925.

Pantazopoulos I, Chalkias A, Mavrovounis G, *et al*. Nasopharyngeal Wash with Normal Saline Decreases SARS-CoV-2 Viral Load: A Randomized Pilot Controlled Trial. *Can Respir J.* 2022; v.2022: 8794127. doi:10.1155/2022/8794127.

Sevinç Gül SN, Dilsiz A, Sağlık İ, Aydın NN. Effect of oral antiseptics on the viral load of SARS-CoV-2: A randomized controlled trial. *Dent Med Probl.* 2022; 59(3): 357-363. doi: 10.17219/dmp/150831

Spinato G, Fabbris C, Costantini G, *et al.* The Effect of Isotonic Saline Nasal Lavages in Improving Symptoms in SARS-CoV-2 Infection: A Case-Control Study. *Front Neurol.* 2021; 12: 794471. doi:10.3389/fneur.2021.794471

Vantarakis A, Velissaris D, Kotsalou C, Oikonomou E, Paraskevas T, Poulas K. First report of reduced severe acute respiratory syndrome coronavirus 2 viral load after nasopharyngeal wash with hypertonic water. *Qeios* 2021; Qeios ID: FIU5K3. <https://doi.org/10.32388/FIU5K3https://www.qeios.com/read/FIU5K3>

Varricchio A, La Mantia I, Brunese FP, Varricchio A, Ciprandi G. Viral shedding in symptomatic patients with mild COVID-19: an experience with nebulized nasal treatment. *J Biol Regul Homeost Agents.* 2021; 35(3): 1155-1157. doi: 10.23812/21-137-L. https://pubmed.ncbi.nlm.nih.gov/34233453/

Yilmaz YZ, Yilmaz BB, Ozdemir YE, *et al*. Effects of hypertonic alkaline nasal irrigation on COVID-19. *Laryngoscope Investig Otolaryngol.* 2021; 6(6): 1240–7. doi: 10.1002/lio2.686

Zarabanda D, Vukkadala N, Phillips KM, et al. The Effect of Povidone-Iodine Nasal Spray on Nasopharyngeal SARS-CoV-2 Viral Load: A Randomized Control Trial. *Laryngoscope.* 2021; 10.1002/lary.29935. doi: 10.1002/lary.29935. https://onlinelibrary.wiley.com/doi/full/10.1002/lary.29935

Zou R, Peng L, Shu D, *et al.* Antiviral Efficacy and Safety of Molnupiravir Against Omicron Variant Infection: A Randomized Controlled Clinical Trial*. Front Pharmacol.* 2022; 13:939573. doi: 10.3389/fphar.2022.939573.

**References of not tabulated studies:**

Detailed list of rejected studies can be obtained on request.

**[References to Legend of Table S3]**

Abdollahi A, Shakoori A, Khoshnevis H, et al. Comparison of Patient-collected and Lab Technician-collected Nasopharyngeal and Oropharyngeal Swabs for Detection of COVID-19 by RT-PCR. Iran J Pathol. 2020;15(4):313-319. doi:10.30699/ijp.2020.127312.2387

Agaoglu NB, Yildiz J, Akgun Dogan O, et al. COVID-19 PCR test performance on samples stored at ambient temperature. *J Virol Methods*. 2022; 301: 114404. doi:10.1016/j.jviromet.2021.114404. <https://www.ncbi.nlm.nih.gov/pmc/articles/PMC8673954/#!po=31.2500>

Jamal AJ, Mozafarihashjin M, Coomes E, et al.; Toronto Invasive Bacterial Diseases Network COVID-19 Investigators. Sensitivity of midturbinate versus nasopharyngeal swabs for the detection of severe acute respiratory syndrome coronavirus 2 (SARS-CoV-2). *Infect Control Hosp Epidemiol.* 2021; 42(8): 1001-1003. doi: 10.1017/ice.2020.1326.

Kinshella MW, Tilley P, Al-Rawahi GN, et al. Evaluation of observed and unobserved self-collection of saline gargle samples for the detection of SARS-CoV-2 in outpatients. *Diagn Microbiol Infect Dis.* 2022; 102(2): 115566. doi: 10.1016/j.diagmicrobio.2021.115566.

McNaughton CD, Adams NM, Hirschie Johnson C, Ward MJ, Schmitz JE, Lasko TA. Diurnal Variation in SARS-CoV-2 PCR Test Results: Test Accuracy May Vary by Time of Day. J *Biol Rhythms.* 2021; 36(6): 595-601. doi: 10.1177/07487304211051841.

Pinninti S, Trieu C, Pati SK, et al. Comparing Nasopharyngeal and midturbinate nasal swab testing for the Identification of Severe Acute Respiratory Syndrome Coronavirus 2. *Clin Infect Dis*. 2021; 72(7): 1253-5. doi: 10.1093/cid/ciaa882.

Zhuang X, Wang W, Borrmann H, et al. Time-of-Day Variation in SARS-CoV-2 RNA Levels during the Second Wave of COVID-19. *Viruses.* 2022; 14(8): 1728. doi:10.3390/v14081728.

Appendix C. Effect on viral shedding

**Table S4. Clinical studies with saline irrigation (SI) or saline nasal spray (smaller volumes) in COVID-19 by disease severity and special target groups*.*** For study retrieval, see supplement section A. Studies are listed by

(A) Prophylaxis (also including 1 study with SARS-CoV-1 infection);

(B) Treatment and disease severity: (B.1.) OUTPATIENTS: listed by (a) Mild-to-moderate COVID-19; (b) Moderate COVID-19 + high respiratory burden; (c) 55-plus; (d) Mild COVID + Smell & Taste disturbances; (B.2.) HOSPITALISED: listed by (a) no ARDS; (b.) ARDS patients; (c) mixed.

| ***Type of participant/ patient***  ***First author***  ***Reference trial registry*** | | ***Treatment(s)***  ***Comparator ^a^*** | ***Trial design***  ***N=Number of patients*** | ***Clinical outcomes (symptoms)*** | | |
| --- | --- | --- | --- | --- | --- | --- |
|  |  |  |  | ***Parameter*** | ***Evidence for SI*** | ***Comments*** |
| **A. PROPHYLAXIS** | | | | | | |
| [SARS-CoV-1 study]  COVID-clinic, upon high-risk contact, China  *Liu et al. 2009* | | Prophylaxis  Nasal SI after contact with infectious insult | Case control study in COVID-ward  N=193 SI  N=284 Ctls  Cases: N=51  Ctls: N=426 | Response to acute prophylaxis:  % PCR-positive HCWs | - SI: (n=9/193): 4.7% infected - Ctls: (n=42/284): 14.8% infected (*P*<0.00002 univariate analysis)   In multivariate analysis not performing nasal rinse was associated OR of 2.41 of acquiring SARS (0.98-5.93) (*P*=.056) | SARS-CoV  Many parameters co-assessed, such as various types of mask wear |
| During first wave:  Physicians/care personal –Dept infectiology, Dept Cardiology, Primary care, United States  *Parviz et al. 2020* | | Prophylaxis  Daily nasal SI + gargling, also eye rinse  At least 3 x daily | Observational feedback from 3 clinical practices + environment, exposed to patients | Response to prophylaxis:  PCR-positivity HCWs | - No infection among saline users - Infected: saline non-users (4 other cardiologists; receptionist, 2 other providers in these exposed practices) | Anecdotal feedback  Author and family physician also report to have observed success of the saline protocol in many patients with COVID-19 infection |
| During first wave:  Front-line HCWs (nurses, physicians), Mexico  *Gutiérrez-García et al. 2021*  RPCEC00000357 in the Cuban Public. Registry of Clinical Trials | | Naso- and oro-pharyngeal SI with neutral electrolysed saline (ES), Esteriflu nose spray + Estericide mouthrinse ^b^  3x daily, 4 weeks add-on to PPE | Randomised controlled study  N=84 ES  N=79 Ctls | Response to prophylaxis:  % Staff PCR-positive | - SI: 1.2% - Ctls: 12.7%   (*P*= 0.0039)  Differences not due to comorbidities: 29.8% in the saline group, 16.5% in Ctls | ES containing 15 ppm of chlorine^b^: role unknown (no normal saline control group). Protocol states ‘saline’ as intervention while 15 ppm is below the targeted concentration in the electrolysis patent.^b^ Currently, no comparative studies in respiratory disease (Chen et al. 2022). |
| 55-plus, high BMI  (Mean 65 years, BMI 30,3)  *Baxter et al. 2022*  ClinicalTrials.gov NCT04559035 | | 0.9% SI(+PVI or NaHCO_3_)  2x/day, 14 days  [NAVAGE or Neilmed Sinus Rinse]  Started within 24 hrs after PCR-test | Open prospective  SI N=78  Control data from meta-analysis | Household transmission | 12.7%(by intention-to-treat) had household contacts positive at >one day after enrolment, compared to 18.8% meta-analysis | See Section B of this Table |
| During Omicron Wave:  HCWs in COVID-19 designated hospital, Shenzhen, China  *Cao et al. 2022* | | Nasal SI daily as innovative treatment added to PPE  If intensive occupational contact: + Molnupiravir, 5 days + Isolation 3-5 days depending on extent of exposure | Open prospective  Obligatory SI use as part of protocol to reach  Zero-COVID-19- strategy  N=not stated (see results | Response to prophylaxis:  % HCWs PCR-positive | Zero infections among HCWs  Liu et al 2022: hospital caring by HCWs for 1,739 patients with COVID-19 admitted to the isolation wing as of February 28, 2022, and 1,836 outpatients and 832 inpatients daily in original wing | Letter communication  Strict (video) control of PPE donning & removal: nasal SI prophylaxis is combined with molnupiravir course in case of occupational exposure and 3-5 days isolation |
| **B. AS PART OF TREATMENT INTERVENTION/STANDARD OF CARE (SOC)** | | | | | | |
| ***B.1. OUTPATIENTS*** | | | | | | |
|  | **a. Mild-to-Moderate COVID-19** | | | | | |
| Mild to moderate COVID-19 (first wave)  *Jadhav et al. 2021* | | Nasal washing + gargling 'salty water'  Online instructed  10x/day, 14 days or until better  Initiated within 24 hrs of test | Randomised controlled  N=35 SI  N=36 Ctls | Symptom relief (scored 1-10 in diary)  Hospitalization rates | SI > Ctls (*p<0.05*) for headache, postnasal drip, anosmia, sinusitis, sore throat, body ache, dry cough (*P<0.05*)  SI (37%) < Ctls (44%) – Not significant | Small study  Self-diagnosis,  Low adherence to SI  Unusually high hospitalization rates: main reason: oxygen <93 (53%), fever not subsiding (39%), breathlessness (17%), cough aggravation (7%) |
| Mild COVID-19    *Kimura et al.2020*  *Esther et al. 2022*  NCT04347538 | | SI 3%, self-managed  Vs Ctls  Vs SI 3% + detergent  2x/day, for 21 days  Interim analysis  Full analysis | Randomised controlled  N=15 (14)/group  N=24/group | Symptoms  (WURS-21)^c^  Time to resolution of individual symptoms  Total WURS score^c^  Viral shedding | SI=SI+shampoo > Ctls  (Kimura):  SI > Ctls for resolution of nasal congestion (5 vs 14 days) (*P=0.04*), headache (3 vs 11 days) (*P=*0.02); trend for cough (*P=*0.19) and fatigue (*P=*0.17); SI > SI+shampoo  (Esther): figure reveals faster WURS evolution with SI, yet SI= Ctls (not significant after controlling for Day 1 symptom score, RNA P and other covariates)  “No evidence of SI-mediated spread of virus to olfactory epithelia”  Mid-turbinate viral load: see Suppl. Table S3 | Kimura: small patient sample, well balanced  Esther: larger sample, yet only cumulated WURS score of highly variable symptom scores (while no baseline score); censoring patients unclear (dots in Figure?) |
| Mild-to-moderate COVID-19    *Spinato et al. 2021*  Treviso and Belluno provinces ethic vote: 871/CESC | | 0,9%, 250 mL [Lavonase]  Once daily, 12 days | Prospective open Matched controls  N=68 SI  N=72 Ctls | Symptoms  Ad hoc questionnaire  Day0, Day10  Viral shedding/ clearance | SI > Ctls:   - scores and % SI patients improving blocked nose (*P*<0.001), sneezing (*P*<0.001) vs baseline - % Ctls worsening blocked nose (*P*<0.001), sneezing (≤ 0.05), runny nose (*P*≤ 0.001)   SI=Ctls for altered smell or taste, watery eyes, pressure in ears  SI better than Ctls: see Suppl. Table S3 | Statistical intergroup analysis of changes not reported  90% declared lavages were simple to use  70% declared they were satisfied |
| Mainly (81.3%) symptomatic COVID‑19  *Chalageri et al. 2022*  CTR India /2020/09/027687 | | 0.9% gargling  3x/day, 21 days  Vs PVI 0,5% 3x/day  Vs Steam 3x/day  Vs Ctls (= Antipyretic, antibiotics, zinc supplements, and  Vitamin C) | Randomised, comparator (investigator blinded by sealed envelopes)  N=80  N=20/group | Symptom recovery: sum of symptoms; recovery=sum reaching 0  Hospitalization (reported)  Viral shedding | SI > other interventions:   - earlier recovery with SI vs other intervention groups (*P*=0.01) - symptom relief seen for fever, cough, malaise, and nasal congestion with SI   SI: N=1 as not improving  PVI: N=2 developed breathing difficulty  SI=other interventions, see Suppl. Table S3 | Gargling alone may relieve symptoms, yet may not be as sufficient as nasal SI, as to prevent hospitalization and to reduce NP viral loads |
| Adults PCR-positive (Age ~44 years)    Za*rabande et al. 2021*    NCT04347954 | | 0.9% nasal spray, 2 sprays/ nostril (smaller volume as placebo)  4x day, 5 days  Vs PVI 0,5%  Vs PVI 2.0% | Randomised, double-blind, comparator  N=11 SI (placebo)  N=11 PVI 0.5%  N=14 PVI 2.0% | Symptom relief  Hospitalization  Viral shedding  AEs | SI > PVI 2%=PVI 0,5%  Not significant vs comparator   - Significant improvements in fever, chills, fatigue, and congestion v baseline with SI (*P*<0.05) - 82% improved with SI > 77% PVI 0,5% > 73% with PVI 2%   SI=PVI: 0%  SI=PVI: see Suppl. Table S3     - More nasal burning and pain with PVI than nasal SI: (PVI 2%: 93%> 28% PVI 0.5% > SI 17%) - with SI also less headache, sneezing and no bleeding observed | Not powered to detect differences in symptom relief |
| Mild COVID-19  *Soler et al 2022*  CONABIOS code 036-2020 in Santo Domingo, Dominican Republic, also registered as NCT04610801 | | 0.9% nasal spray,  2 pumps/nostril (smaller volume than SI, reference)  Every 3 hrs, 3 days, followed by every 6 hrs, 14 days  Vs Xylitol spray (containing 0.85% NaCl + flavonoids) | Randomised, double-blind, comparator  N=50  (N/group not disclosed) | Symptom recovery VAS, DSS; score for smell:  change from baseline  Hospitalization | More patients recovered with SI than comparator for nasal congestion; % patients still with symptom:   - Day 3: 41.7% vs 73.1 (*P*=0.025) - Day 7: 17.4% vs 50.0% (*P*=0.017), resp.   = : SI = comparator for overall VAS (*P*=0.124), DSS (*P*=0.448) and sense of smell (*P*=0.667)  0% in both groups | Bias: the study claims persistent anosmia only to be observed with saline, yet this is not confirmed in the Figure of smell scores (comparable for both groups at Day 28) |
| Mild COVID-19 (<37,5°C) + smell, taste    *Varricchio et al. 2020* | | SI 3%, HMW-Na hyaluronate, xylitol (Alumeb)  + SOC: tobramycin/ lincomycin | Open prospective  (No Ctls)  N=76 | Evaluation Day7  Hospitalization  Viral shedding | Symptoms resolved Day 7  0%  Day 10 : see Suppl. Table S3 | Letter communication (little details) |
| Mostly mild symptomatic COVID-19  *Zou et al 2022*  ChiCTR2200056817 | | Molnupiravir (800 mg) add-on to daily basic treatment (=nasal SI + Chinese flue medicine 2x/ day)  Vs daily nasal SI + Chinese flue medicine, 2x/ day | Randomised, cohort study,  N=107  N= 31 SI  N=77 Molnupiravir  add-on SI | Symptoms recovery  Viral shedding/ clearance  AEs | Molnupiravir add-on = Daily nasal SI   - Median time to alleviate COVID-19 symptoms: 5 days add-on vs. 7 days without, *P*=0.499) - Duration of fever reduced by 2 days add-on Vs without (1 day vs. 3 days, *P*= 0.096).   Faster viral clearance with combination: see Suppl. Table S3  AEs: N=3 in molnupiravir group, N=0 on SI | No information on duration of symptoms prior to enrolment (potentially biasing results)  No details on nasal saline irrigation practices |
|  | **b. Moderate COVID-19 + large respiratory burden** | | | | | |
| Adults (Age ~35, 27-45 years,  + large respiratory burden    *Ezer et al. 2022*  ClinicalTrials.gov NCT04435795 | | Nasal 0.9% saline placebo drops (placebo)  2x/day, 14 days  (=smaller volumes)  Vs ciclesonide inhaler (600 μg 2x/ day) OR intranasal (200 μg daily) | Randomised, double-blind, comparator  N=98 SI  N=105 ciclesonide | Symptom resolution  Hospitalisation | At day 7:  Saline (35%) = Comparator (40%)  At day 14:  Saline 58% = Comparator (66%)  SI: 3/98 (3%) < Comparator 6/105 (5.7%) |  |
|  | **c. COVID in patients aged >55 years** | | | | | |
| 55-plus, high BMI  (Mean 65 yrs, BMI 30,3)  *Baxter et al. 2022*  ClinicalTrials.gov NCT04559035 | | 0.9% SI(+PVI or NaHCO_3_)  2x/day, 14 days  [NAVAGE or Neilmed Sinus Rinse]  Started within 24 hrs after PCR-test | Open prospective  SI N=78  (Randomised for device)  Matched controls  (50-plus CDC-database same period)  N= 2,962,541 | Symptom resolution, severity  Hospitalisation (+ mortality)  Household transmission  AEs: | Symptoms in >50% of patients: fever, muscle aches, congestion, and headache:   - Symptom resolution more likely for those reporting twice daily SI than once *(P=.0031)* regardless of additive - symptoms resolved in all but 8 (12.9%) over 14 days   SI 1/78 (1.28%, no mortality) << CDC Ctls (9.47% + additional 1.5% mortality)  12.7% (by intention-to-treat) had household contacts positive at >one day after enrolment, compared to 18.8% meta-analysis  11 patients complained of discomfort or spotty epistaxis, with four discontinuing SI | Navage system = pressurized system (allows to collect rinse fluid)  No differences in outcomes by irrigation system or additive |
|  | **d. Mild COVID + Smell & Taste disturbances** | | | | | |
| Young adults with olfactory dysfunction/anosmia > 3 days  (84%-85% also ageusia)    *Rashid et al. 2022*  ClinicalTrails.gov, NCT04569825 | | Nasal saline 0.9% (placebo drops)  Vs betamethasone  (BM) 1 mg/mL drops  3x/day until recovery for < 1 month | Randomised, double-blind, comparator  N=138/group | Time of  recovery from anosmia | SI=BM:   - 84% - 82% recovered from anosmia within 30 days, respectively - Median recovery time=7 days, median recovery time from onset symptoms=13 days in both groups; Hazard ratio: BM shows slower recovery from anosmia than SI, yet not significant (*P*=0.31) - Younger participants recover faster than older participants in both groups - Co-presence of ageusia affects the BM outcome, but not the SI outcome | Age and duration of anosmia before intervention highly impact recovery, yet not outcome  Results contrast to promising outcomes of initial open prospective studies with steroids  -> Saline more than a placebo? |
| Recovered PCR-negative patients with smell disturbances (severe microsmia or anosmia), symptoms already for 2 weeks    *Kasiri et al. 2022*  Iranian Registry of Clinical Trials (Code: IRCT20190804044429N6) | | Nasal saline 0.9%  (placebo spray, 0.5 mL per dose)  Vs mometasone (MM)  furoate 0.05% (100µg)  2x/days, 4 weeks  + olfactory training | Randomised, double-blind, comparator  N=80 (77 analysed)  N=40/group | Change in smell disturbance severity | SI = MM:   - Change in VAS from baseline - Change in Smell Test (Iran-SIT*) score   Severity distribution at study end in favour of MM (*P*<0.001)  One hospitalised in both groups (1/40), determined as lost to follow up (SI only started after 2 weeks, unclear if hospitalized for COVID and at what stage) | Patient groups not 100% matched (More headache in SI than Ctls (P=0.017)  No information on pre-existing symptom duration which may have affected end results of severity/ resolution distribution |
| Taste/smell disturbances  *Varricchio et al. 2020* | | 3.0% SI + nebulising  [Rinowash Sicom + Nebula aerolizer]  2x/day, 7-10 days | Open prospective  (No Ctls)  N=46 | Recovery smell and taste disturbances | Anosmia/ageusia: all recovered normal smell and taste between 10 and 20 days  Cacosmia between 20 and 40 days after treatment onset | Letter format  No AEs |
| Taste/smell disturbances    *Yildiz et al. 2021*  Afyonkarahisar Health Science University Clinical Research Ethic Committee (05.03.2021–2021/2) | | SI (hypertonic) 10 mL  SI + triamcinolone acetate (2*2 puffs/each nose  2x daily, 1 month  Vs controls | Randomised, comparator  N=150  (50/group) | Self-rated symptom score (1-10= no-full odour)  Subjective  Olfactory Capability | SI alone: no significant effect on scores (smell/taste disturbances), duration or subjective olfactory capability  SI + steroid: significantly better than Ctls and SI alone on scores (*P*=0.018, *P* =0.033), duration (*P*=0.029, *P* =0.022) and subjective olfactory capability (*P*=0.05) |  |
| ***B.2. HOSPITALISED*** | | | | | | |
|  | **a. No ARDS** | | | | | |
| Pneumonia (-ARDS); discharged + treated as outpatients  *Chatterjee et al. 2020*  CTRI/2020/08/027465 | | SI 0.9%, 25 mL spray bottle  3x 🡪 up to 8x/day,  7 days  [*For video instructions, see Legend*] | Dose-finding  Matched CTLs  N=61 SI  N=64 Ctls | CT scan severity score  % Worsening  Viral shedding | Lung severity score on CT scan improved or static in 31/36 (91%) with SI versus 14/22 (63%) with Ctls  only 3 worsening over 7 with SI days versus 8 worsening over 7 days among controls (*P*=0.028)  Dose-finding: see Suppl. Table S3 | Methods used: manual pressurising spray bottle |
| Moderately symptomatic COVID-19 (-ARDS)  *George et al. 2022*  CTRI/2020/09/028084 | | 0.9% nasal spray (low volume, reference)  Vs CaCl_2_-0.39% NaCl mixture (FEND-inhaling system):  3x/ day, for 3 days, add-on to SOC (azithromycin, paracetamol) | Random controlled study  N=20 (20) SI  N=20 (17) CaCl_2_  Results analysed after exclusion pts taken up in ICU  Patient data in supplement show:  N=20 SI  N=33 CaCl_2_  N=10 Ctls (no data on discharge) | ICU uptake  Symptom relief  Oxygenation | SI (0% in ICU) Vs CaCl_2_ (3/20 in ICU = 15%)  After exclusion ICU patients:   - FEND (rising oxygen saturation) = saline (no change with saline, yet pO2 was 97% with NS throughout study) (*P*=0.533) - % Needing steroids or antibiotics: FEND = saline (inconsistencies between text, Figure and legend) - Symptoms, at discharge Day 5: FEND 86% without symptoms, saline 0% without symptoms.   Yet supplement indicates:   - Emergency care/Escalation/in hospital :   - 4/20 FEND = 20% (all FEND-population data:8/40 =20%)   - 1/20 SI= 5% (=ITT)   - 1/10 Ctls = 10% | Inconsistencies in protocol and analysis:  - 3-5 days of assessment?  - data with NS in all (none in ICU), yet failures with CaCl_2_ excluded  - Need for oxygenation was set as SpO2<95%, yet oxygen was 97% on average with S  The supplement on the total patient sample confirms the higher risk of ICU or treatment escalation in the total FEND-treated sample.  Other study on tolerability reported a high incidence of AEs in the probants (e.g. cough in 50%, Edwards 2020 ) |
| Moderate pneumonia, moderate (<25% lung involvement) (-ARDS)    *Yilmaz et al. 2021*  Scientific Research Projects Coordination Unit of Istanbul University-Cerrahpasa, Project number: 34932 | | HCQ+ SI (2.3%, pH 10) spray  one puff/nostril  4x/ day, for 7 days  *vs* HCQ | Randomised, controlled  N=30 SI  N=30 Ctls | Hospitalized  Viral shedding | SI (0% in ICU) Vs Ctls (3/20 in ICU = 10%)  See Suppl. Table S3 | Hospitalization due to development dyspnoea, tachypnoea, respiratory distress, SpO2<95%  No epistaxis, smell loss, and burning |
| COPD patients hospitalized in rehabilitation stage of COVID-19  *Titova et al. 2022* | | 7% SI+0.1% sodium hyaluronate nebulization (inhaling) | Randomised, controlled  N=25/group | Symptom severity  Sputum discharge  Quality-of-life | - SI: significant decrease in severity of cough, and sputum discharge - Tendency to reduce shortness of breath and to improve quality of life | No serious adverse events  [Russian: only abstract available] |
|  | **b. ARDS patients** | | | | | |
| ARDS, needing oxygen, but no invasive oxygen support  *Voshaar et al. 2020* | | Saline neb (0.9%) daily  At least 1x/day  add-on to SOC | Open prospective  N=60 | Needing MV:  Mortality (1st wave)  Transmission | - Need of MV:3/60 (5%) (comorbidities) - No secondary spreading   1/60 (1,7%) died (multiple co-morbidities)  No dissemination/transmission to HCWs | Reporting in German |
| ARDS, developing oxygen desaturation, 74.3% on supplemental oxygen at baseline    *Salva et al. 2020*  NCT04382768 | | Saline neb (3%)  5 mL 3x/day, at least 15 min, 3x/ day  at least 1 day until discharge  + Sodium ibuprofenate (IB) 50 mg | Prospective open  Matched controls  N=383 (56 on MV) saline neb + IB  N=195 (21 on MV) Ctls (no use) | NEWS2 score  ALOS (average length of hospital stay)  Mortality (1st wave) | Worsening NEWS2 scores prior to treatment.  Nebulised HS+IB was associated with:   - rapid improvement in hypoxia (mean News2 score, RR and sO2) and vital signs (25 patients never required oxygen supplementation) - rapid reversal of deterioration in oxygenation and of NEWS2 scores after initiation of NS+IB, regardless MV   Shorter ALOS: SI+IN: 11.5 ± 0.3 days vs 13.3 ± 0.9 days for Ctls (*P*<0.01) if no MV; similar benefit if MV  Without MV:   - SI: 10.7% vs no use: 19.5% (*P*<0.03)   With MV:   - SI: 19.6% vs no use: 86,7% (*P*<0,001) | Role of ibuprofen in nebulization procedure to be investigated |
| Severe ARDS, ventilated patients  *Delić et al. 2022*  clinicaltrials.gov NCT 04755972 | | Saline neb (5%) 2x/day  Vs Ctls (no inhalation)  Vs nebulized  N-acetylcysteine  Vs nebulized  sodium bicarbonate  (8.4%) | Randomised controlled, 4 arms  N= 53 Ctls  N= 39 HS  N= 42 N-acetyl cysteine  N=42 bicarbonate | % MSRA  Mortality  AEs | 14.3% with SI (= comparators) < 34.6% with Ctls (*P*=0.05)  40.5% with SI, 47.6% with sodium bicarbonate, 53.8% with N-acetylcysteine  < 59.5% with Ctls [not significant for the inhalation group (all treatments) vs Ctls]  No significant AEs with HS; bronchospasm with N-acetylcysteine (N=1, 2.4%) and with sodium bicarbonate (N =1, 2.6%) | Study not setup (sample size not powered) to detect differences in mortality |
|  | **c.Mixed** | | | | | |
| Hospitalised in COVID-19 designated hospital (2-wong model) - Omicron  *Cao et al. 2022*  *Liu et al. 2022* | | Daily nasal normal SI as innovative treatment add-on to antiviral treatment/oxygenation if needed and isolation | Open prospective (new innovative strategy in zero-COVID-policy) | Mortality (Omicron) | No deaths due to COVID-19 (Omicron) | Letter and communication with no details on treatment, % patients with ARDS and ventilation/ICU requirements |

Abbreviations: IS=isotonic saline; HS= hypertonic saline; Neb=nebulized saline; Ctls=Controls; AEs = adverse events; vs = versus/compared with.

Comparators or additives: BM=betamethasone; ES = electrolysed saline; CaCl_2_=Calcium chloride mixture (FEND); HCQ=hydroxychloroquine; IB= ibuprofen; MM= mometasone furoate; PVI= Polyvidone iodine; SI= (oronasal) saline irrigation. See also Table S3.

^a^ Also studies using saline nasal spray are included, if the spray is used at least twice daily. If saline is just administered by a metered dose inhaler, this is not considered a study allowing to address effects of saline (irrigation). Studies using electrolysed saline (irrigation) are not listed, except under section A. Prophylaxis.

^b^ Nasopharyngeal rinses were performed by applying four sprayings (~0.4 ml) of ES (EsteriFlu®) to each nostril using the nasal valve, three times a day. Oropharyngeal rinses were performed by gargling 10 ml of ES (Estericide® Bucofaríngeo), during 60 sec, three times a day. ES = electrolysed isotonic saline generating 0.0015% chlorine, pH 6.0-7.5, which is likely below the virucidal MIC against SARS-CoV-2 (as assessed for the eye by Gessa-Sorroche 2022), observed in vivo. It is below the concentration targeted in the patent. No comparisons withs aline available (Chen et al. 2022). Patent: MX2016013933A [<https://patents.google.com/patent/MX2016013933A/en> - Accessed 15.12.2022]

^c^ WURS-21 = Wisconsin Upper Respiratory Symptom Survey.

^d^ Aggravation Ct scan (Ct); Intensive care rate (ICU); Mechanical Ventilation (MV).

**Video Instructions of SI, as used in the studies**:

*Chatterjee et al. 2020*

https://www.youtube.com/shorts/DuXREn8dML8

<https://www.youtube.com/shorts/yG1ogCe6PX8>

*Parviz et al. 2020*

SWHF-ERNIG protocol.

<https://www.youtube.com/watch?v=1yDgJ80hCoU>

**References of studies tabulated in Table S4 (alphabetical):**

Baxter AL, Schwartz KR, Johnson RW, Kuchinski AM, Swartout KM, Srinivasa Rao ASR, et al. Rapid initiation of nasal saline irrigation to reduce severity in high-risk COVID+ outpatients. *Ear Nose Throat J.* 2022 Aug 25:1455613221123737. doi: 10.1177/01455613221123737

Cao J, Wen M, Shi Y, Huang T, Yi Y, Su Y, et al. How should designated COVID-19 hospitals in megacities implement a precise management strategy in response to Omicron? *Biosci Trend*s. 2022;16(3):242-244. doi:10.5582/bst.2022.01261

Chalageri VH, Bhushan S, Saraswathi S, Ranganath TS, Rani VD, Majgi SM, et al*.* Impact of Steam Inhalation, Saline Gargling, and Povidone-Iodine Gargling on Clinical Outcome of COVID-19 Patients in Bengaluru, Karnataka: A Randomized Control Trial. *Indian J Community Med.* 2022; 47(2): 207-212. doi: 10.4103/ijcm.ijcm_804_21

Chatterjee U, Chakraborty A, Naskar S, Saha B, Bandyapadhyay B, Shee S. Efficacy of normal saline nasal spray and gargle on SARS-CoV-2 for prevention of COVID-19 pneumonia. *Research Square.* Preprint 2021; PPR: PPR277214. doi:10.21203/rs.3.rs-153598/v1

Delić N, Matetic A, Domjanović J, Kljaković-Gašpić T, Šarić L, Ilić D,et al. Effects of Different Inhalation Therapy on Ventilator-Associated Pneumonia in Ventilated COVID-19 Patients: A Randomized Controlled Trial. Microorganisms. 2022 May 28;10(6):1118. doi: 10.3390/microorganisms10061118.

Esther CR Jr, Kimura KS, Mikami Y, Edwards CE, Das SR, Freeman MH, et al. Pharmacokinetic-based failure of a detergent virucidal for severe acute respiratory syndrome-coronavirus-2 (SARS-CoV-2) nasal infections: A preclinical study and randomized controlled trial. *Int Forum Allergy Rhinol.* 2022:10.1002/alr.22975. doi:10.1002/alr.22975

Ezer N, Belga S, Daneman N, Chan A, Smith BM, Daniels SA, et al. Inhaled and intranasal ciclesonide for the treatment of covid-19 in adult outpatients: CONTAIN phase II randomised controlled trial. *BMJ.* 2021; 375: e068060. doi:10.1136/bmj-2021-068060

George CE, Scheuch G, Seifart U, et al. COVID-19 symptoms are reduced by targeted hydration of the nose, larynx and trachea. *Sci Rep. 2022*; 124599

Gutiérrez-García R, De La Cerda-Ángeles JC, Cabrera-Licona A, Delgado-Enciso I, Mervitch-Sigal N, Paz-Michel BA. Nasopharyngeal and oropharyngeal rinses with neutral electrolyzed water prevents COVID-19 in front-line health professionals: A randomized, open-label, controlled trial in a general hospital in Mexico City. *Biomed Rep.* 2022; 16(2): 11. doi: 10.3892/br.2021.1494.

Jadhav RB, Patil SS, Deolekar P, Yadav P. A comparative study to evaluate the use of saline nasal lavage and gargling in patients with COVID-19 infection. *Int J Pram Res. 2022*; 14: 12-17. EMBASE | ID: covidwho-1668051

Kasiri H, Rouhani N, Salehifar E, Ghazaeian M, Fallah S. Mometasone furoate nasal spray in the treatment of patients with COVID-19 olfactory dysfunction: A randomized, double blind clinical trial. *Int Immunopharmacol.* 2021; 98: 107871. doi: 10.1016/j.intimp.2021.107871.

Kimura KS, Freeman MH, Wessinger BC, Gupta V, Sheng Q, Huang LC, et al. Interim analysis of an open-label randomized controlled trial evaluating nasal irrigations in non-hospitalized patients with coronavirus disease 2019. *Int Forum Allergy Rhinol.* 2020; 10(12): 1325-1328. doi: 10.1002/alr.22703

Spinato G, Fabbris C, Costantini G, Conte F, Scotton PG, Cinetto F, *et al.* The Effect of Isotonic Saline Nasal Lavages in Improving Symptoms in SARS-CoV-2 Infection: A Case-Control Study. *Front Neurol.* 2021; 12: 794471. doi:10.3389/fneur.2021.794471

Liu W, Tang F, Fang LQ, et al. Risk factors for SARS infection among hospital healthcare workers in Beijing: A case control study. *Trop Med Int Health.* 2009; 14(s1) :52-9. doi: 10.1111/j.1365-3156.2009.02255.x

Liu X, Cao J, Ji Y, Li T, Zhu Z, Huang T, Lu H. An innovative two-wing model for balancing the demands of inpatients with COVID-19 and general medical service in a designated hospital for COVID-19 in Shenzhen, China. *Biosci Trends.* 2022; 16(2): 163-166. doi:10.5582/bst.2022.01106.

Parviz S, DuncanL, Rabago D. Soap and Water to Hands and Face-Eye Rinse, Nasal Irrigation and Gargling with Saline for COVID-19 with anecdotal evidence. *Rhinology.* 4(4):185-193. doi: 10.4193/RHINOL/21.032

Rashid RA, Zgair A, Al-Ani RM. Effect of nasal corticosteroid in the treatment of anosmia due to COVID-19: A randomised double-blind placebo-controlled study. *Am J Otolaryngol.* 2021; 42(5): 103033. doi:10.1016/j.amjoto.2021.103033.

Salva O, Doreski PA, Giler CS et al. Reversal of SARS-CoV2-Induced Hypoxia by Nebulized Sodium Ibuprofenate in a Compassionate Use Program. *Infect Dis Ther.* 2021; 10(4): 2511-2524. doi: 10.1007/s40121-021-00527-2

Soler E, de Mendoza A, Cuello VI, Silva-Vetri MG, Núñez ZH, Ortega RG et al. Intranasal Xylitol for the Treatment of COVID-19 in the Outpatient Setting: A Pilot Study. *Cureus.* 2022; 14(7): e27182. doi:10.7759/cureus.27182

Titova O.N., Kuzubova N.A., Skliarova D.B., Aleksandrov A.L., Egorova N.V. The effectiveness of combination of 7% hypertonic saline and 0.1% natrii hyaluronas in patients with chronic obstructive pulmonary disease who have suffered a new coronavirus infection. *Meditsinskiy sovet = Medical Council.* 2021; (16):85-91. (In Russ.) doi: 10.21518/2079-701X-2021-16-85-91

Varricchio A, La Mantia I, Brunese FP, Varricchio A, Ciprandi G. Viral shedding in symptomatic patients with mild COVID-19: an experience with nebulized nasal treatment. J *Biol Regul Homeost Agents.* 2021; 35(3): 1155-7. doi:10.23812/21-137-L. https://pubmed.ncbi.nlm.nih.gov/34233453/

Varricchio A, La Mantia I, Brunese FP, Ciprandi , G. Smell recovery in patients with COVID-19: an experience with nebulized nasal treatment. *J Biol Reg Hom Agents*. 2021; 35(2): 683-686. doi: 10.23812/21-28-L.

Voshaar T. COVID-19 Therapie aus Sicht eines Aerosol-Experten. PARI.de - *Artzeportal 28 Juli 2020.* [https://www.pari.com/de/aerzteportal/news/covid-19-therapie-aus-sicht-eines-aerosol-experten](https://www.pari.com/de/aerzteportal/news/covid-19-therapie-aus-sicht-eines-aerosol-experten/) Accessed 10 January 2020

Yildiz E, Koca Yildiz S, Kuzu S, Günebakan Ç, Bucak A, Kah-veci OK. Comparison of the Healing Effect of Nasal Saline Irrigation with Triamcinolone Acetonide Versus Nasal Saline Irrigation alone in COVID-19 Related Olfactory Dysfunction: A Randomized Controlled Study. *Indian J Otolaryngol Head Neck Surg.* 2021: 1-6. doi:10.1007/s12070-021-02749-9.

Yilmaz YZ, Yilmaz BB, Ozdemir YE, Kocazeybek BS, Karaali R, Çakan D, et a*l*. Effects of hypertonic alkaline nasal irrigation on COVID-19. *Laryngoscope Investig Otolaryngol 2021*; 6(6): 1240–7. doi:10.1002/lio2.686

Zarabanda D, Vukkadala N, Phillips KM, Qian ZJ, Mfuh KO, Hatter MJ, et al. The Effect of Povidone-Iodine Nasal Spray on Nasopharyngeal SARS-CoV-2 Viral Load: A Randomized Control Trial*. Laryngoscope*. 2021; 10.1002/lary.29935. doi:10.1002/lary.29935. https://onlinelibrary.wiley.com/doi/full/10.1002/lary.29935

Zou R, Peng L, Shu D, Zhao L, Lan J, Tan G, *et al.* Antiviral Efficacy and Safety of Molnupiravir Against Omicron Variant Infection: A Randomized Controlled Clinical Trial. Front Pharmacol. 2022; 13:939573. doi:10.3389/fphar.2022.939573.

Reference on low FEND tolerability (CaCl_2_) and on HOCl generated by electrolysed saline:

Edwards D, Hickey A, Batycky R, Griel L, Lipp M, Dehaan W, Clarke R, Hava D, Perry J, Laurenzi B, Curran AK, Beddingfield BJ, Roy CJ, Devlin T, Langer R. A New Natural Defense Against Airborne Pathogens. *QRB Discov.* 2020; 1:e5. doi: 10.1017/qrd.2020.9

Gessa-Sorroche M, Relimpio-López I, García-Delpech S, Benítez-Del-Castillo JM. Ácido hipocloroso como antiséptico en la atención al paciente con sospecha de infección por COVID-19 [Hypochlorous acid as an antiseptic in the care of patients with suspected COVID-19 infection]. *Arch Soc Esp Oftalmol.* 2022; 97(2): 77-80. Spanish. doi: 10.1016/j.oftal.2021.01.012.

Chen BK, Wang CK. Electrolyzed Water and Its Pharmacological Activities: *A Mini-Review. Molecules.* 2022; 27(4): 1222. doi: 10.3390/molecules27041222

**References of not tabulated studies:** Detailed list of rejected studies can be obtained on request.

Appendix C. Effect on viral shedding

**Table S5. Rates of hospitalization and risks (Intensive Care Uptake, Pneumonia severity scores, Ventilation, Mortality).**

For studies and references, see Supplements A and B. Studies are listed by (

(1) OUTPATIENTS: using larger volumes of SI in (a) randomised; (b) matched controls; (c) open prospective studies; use of smaller spray volumes of saline (placebo) in (d) randomised, double-blind, comparator studies.

(2) HOSPITALIZED patients: patients without Acute Respiratory Distress Syndrome (ARDS): (a) randomised studies; (b) matched controls; and patients with ARDS: (a) randomised; (b) matched controls; (c) prospective studies.

| ***Type of patient***  ***Reference*** | ***Treatment(s)***  ***Comparator ^a^*** | ***Parameter*** | ***Saline***  ***(SI, Neb, spray)*** | ***Control (no Saline) or comparator*** |
| --- | --- | --- | --- | --- |
| ***1. OUTPATIENTS*** |  | ***Hospitalization Rate (Hr)*** | | |
| **1a. Randomised** | | | | |
| Moderate COVID-19 (first wave)  *Jadhav et al. 2022* | Nasal washing + gargling 'salty water' (online instructed) 10x/day, 14 days | Hr | Hr: 13/35  (37%)  *N.S.* | 16/35  (46%) |
| Mild COVID-19  *Kimura et al. 2020,*  *Esther et al. 2022* | SI 3%, self-managed, 2x/day  Vs Controls  Vs SI 3% + detergent | Hr | 0/24  (0%) | 0./24 – 0/24  (0%) |
| Taste/smell disturbances  *Yildiz et al.* | SI (hypertonic)  SI + triamcinolone acetate  Vs controls | Hr | 0/50  (0%) | 0/50 - 0/50  (0%) |
| **1b. Matched controls** | | | | |
| 55-plus, high BMI  *Baxter et al.* | 0.9% SI (+PVI or +NaHCO_3_) 2x/day, 14 days  [NAVAGE or Neilmed Sinus Rinse]  Comparator: 50-plus CDC data base | Hr | 1/79  (1.3%)  (*P*<*0006*) | CDC  (9.5%)  [+ death 1.5%] |
| Mild-to-moderate COVID-19  *Spinato et al.* | 0,9%, 250 mL, once daily, 12 days  [Lavonase] | Hr | 0/68  (0%) | 0/72  (0%) |
| **1c. Open prospective** | | | | |
| Mild COVID-19 (<37,5°C)  *Varricchio et al.* | SI + neb 0.9%, 2x/day, 7 days  [Rinowash Sicom + Nebula aerolizer ] | Hr | 0/76  (0%) | - |
| Mild COVID-19 (<37,5°C) + smell, taste  *Varricchio et al.* | SI hypertonic (3%NaCl) , HMW-Na hyaluronate, xylitol (Alumeb) | Hr | 0/42  (0%) | - |
| Taste/smell disturbances  *Varricchio et al.* | SI + neb 0.9%, 2x/day, 7 days  [Rinowash Sicom + Nebula aerolizer] | Hr | 0/42  (0%) | - |
| **1d. Randomised, double-blind, comparator *(smaller volume sprays)*** | | | | |
| Adults PCR-positive (Age ~44 years)  *Zarabande et al.* | Nasal spray SI 0.9%, 2 sprays/nostril, 4x day, 5 days  Vs PVD-I 0,5%  Vs PVD-I 2.0% | Hr | 0/11  (0%) | 0/11 – 0/14  (0% - 0%) |
| Adults (Age ~35, 27-45 years, large respiratory burden  *Ezer et al.* | Metered dose inhaler + nasal saline 0.9%, 2x/day, 14 days  Vx ciclesonide | Hr | 3/98  (3.1%) | 6/105  (5.7%) |
| Young adults with olfactory dysfunction (Age: 29, 23–37 years).  *Al Rashid et al.* | Nasal saline 0.9%  Vs betamethasone  3x/day until recovery for a maximum of one month | Hr | 0/138  (0%) | 0/138  (0%) |
| ***2. HOSPITALISED*** | ***Treatment(s)***  ***Comparator ^a^*** | ***Intensive race risk ^b^*** | | |
| **2a. Randomised, no ARDS** | | | | |
| Mildly symptomatic COVID-19 (-ARDS)  *George et al.* | SI 0.9% spray, 2 large puffs in each nostril, 3x/ day, for 3 days, add-on to SOC (azithromycin, paracetamol)  Vs CaCl2 mixture (FEND) | ICU:  ICU + escalation | 0/20  (0%)  *N.d.*  1/20  (1/20 ITT)  (5%) | 3/20  (15%)  4/20  (8/40 ITT)  (20%) |
| Hospitalized patients with pneumonia  *Pantazopoulos et al.* | SI 0.9% 10 mL per nostril, every 4 hrs for 16 hours  *Bias by age possible due to older age in the control group | Oxygen support  ICU =  Mortality | 2/24  (8.3%)  N.S.*  0/24  (0%)  N.S. | 6/26  (23%)  3/26  (11.5%) |
| Moderate pneumonia, moderate (<25% lung involvement) (-ARDS)  *Yilmaz et al.* | HCQ+ SI (2.3%, pH 10) spraying, one puff/nostril 4x/ day, for 7 days  *vs* HCQ | ICU | 0/30  (0%)  *N.d.* | 3/30  (10%) |
| **2b. Matched controls, no ARDS** | | | | |
| Pneumonia (-ARDS)  *Chatterjee et al.* | SI 0.9%, 25 mL spray bottle  3x 🡪 up to 8x/day, 7 days | CT-scan worsening:^b^  PP [ITT] | 3/34 - [3/64]  (8.8% [4,9%])  *P=0.028* | 8/22 - [8/64]  [36.4 - (12.5%)] |
| **2c. Randomised, ARDS patients** | | | | |
| Ventilated severe ARDS  *Delić et al.* | Saline neb (5%) 2x/day | % MSRA  Mortality | 14.3%  *P = 0.05*  40.5%  N.d. (N.P.) | 34.6%  59.5% |
| **2d. Matched controls, ARDS patients** | | | | |
| ARDS, oxygen desaturation, 74.3% on supplemental  oxygen at baseline  *Salva et al.* | Saline neb (3%), 5 mL 3x/day, at least 1 day until discharge (after 11-13 days on average)  (+ Sodium ibuprofenate 50 mg) | Mortality  -MV  Mortality  +MV | 41/383  (10.7%)  *P<0.04*  11/56  (19.6%)  *P<0.001* | 36/195  (18.4%)  17/21  (80.09%) |
| **2e. Prospective open, ARDS patients** | | | | |
| ARDS, needing oxygenation, but no invasive oxygenation, first wave)  *Voshaar et al.* | Saline neb (0.9%) daily | MV  Mortality ^e^ | 3/60  (5%)^d^  1/60  (1.7%)^e^ | [32.3%]^d^  [17.9%]^d^ |

^a^ HCQ = hydroxychloroquine; PVD-I= Polyvidone iodine; CaCl_2_=Calcium chloride mixture (FEND); SI= (oronasal) saline irrigation; Neb = nebulized saline; N.d. = not determined; N.P.: study sample was not powered to detect significant differences

^b^ Aggravation Computed Tomography scan (CT) (PP: calculated per protocol having undergone a repeated Ct scan, ITT: calculated intention-to-treat on the complete sample irrespective whether or not having undergone a repeat CT scan; Intensive care rate (ICU); Mechanical Ventilation (MV)

^e^ Patients had multiple co-morbidities; n=1 end-of-life patient

^d^ Published rates in Germany for the same period: Hobohm L, Sagoschen I, Barco S,et al. Trends and Risk Factors of In-Hospital Mortality of Patients with COVID-19 in Germany: Results of a Large Nationwide Inpatient Sample. *Viruses.* 2022; 14(2): 275. doi: 10.3390/v14020275.

1. Cazzola M, Ora J, Bianco A, Rogliani P, Matera MG. Guidance on nebulization during the current COVID-19 pandemic. *Respir Med.* 2021; 176: 106236. doi: 10.1016/j.rmed.2020.106236. [↑](#footnote-ref-1)
2. International Pharmaceutical Federation (FIP). Cold, flu and sinusitis. Managing symptoms and supporting self-care. A handbook for Pharmacists. 2021. <https://www.fip.org/file/5089>. [↑](#footnote-ref-2)
3. King D, Mitchell B, Williams CP, Spurling GK. Saline nasal irrigation for acute upper respiratory tract infections. *Cochrane Database Syst Rev.* 2015; 20: 2015(4):CD006821. doi: 10.1002/14651858.CD006821 [↑](#footnote-ref-3)
4. Orlandi RR, Kingdom TT, Smith TL, Bleier B, DeConde A, Luong AU, et al. International consensus statement on allergy and rhinology: rhinosinusitis 2021. *Int Forum Allergy Rhinol.* 2021; 11(3): 213-739. doi: 10.1002/alr.22741. Erratum in: Int Forum Allergy Rhinol. 2022 Mar 11;: PMID: 33236525. <https://onlinelibrary.wiley.com/doi/10.1002/alr.22741>. [↑](#footnote-ref-4)
5. Rabago D. Nasal Irrigation to Treat Acute Bacterial Rhinosinusitis. *Am Fam Physician.* 2005; 72(9): 1661-1663. [↑](#footnote-ref-5)
6. Ramalingam S, Graham C, Dove J, Morrice L, Sheikh A. A pilot, open labelled, randomised controlled trial of hypertonic saline nasal irrigation and gargling for the common cold. *Sci Rep*. 2019; 9: 1015. https://doi.org/10.1038/s41598-018-37703-3. [↑](#footnote-ref-6)
7. Singh S, Sharma N, Singh U, Singh T, Mangal DK, Singh V. Nasopharyngeal wash in preventing and treating upper respiratory tract infections: Could it prevent COVID-19? *Lung India.* 2020; 37(3): 246-251. Doi: 10.4103/lungindia.lungindia_241_20. [↑](#footnote-ref-7)
8. Wise, S. K., Lin, S. Y., Toskala, E., Orlandi, R. R., Akdis, C. A., Alt, J. A., Azar, A., Baroody, F. M., Bachert, C., Canonica, G. W., Chacko, T., Cingi, C., Ciprandi, G., Corey, J., Cox, L. S., Creticos, P. S., Custovic, A., Damask, C., DeConde, A., DelGaudio, J. M., … Zacharek, M. . International Consensus Statement on Allergy and Rhinology: Allergic Rhinitis. *International forum of allergy & rhinology.* 2019; 8(2):108–352. https://doi.org/10.1002/alr.22073. [↑](#footnote-ref-8)
9. Petersen E, Koopmans M, Go U, Hamer DH, et al. Comparing SARS-CoV-2 with SARS-CoV and influenza pandemics. *Lancet Infect Dis.* 2020; 20(9): e238-e244. doi: 10.1016/S1473-3099(20)30484-9. [↑](#footnote-ref-9)
10. Balmforth D, Swales JA, Silpa L, et al. Evaluating the efficacy and safety of a novel prophylactic nasal spray in the prevention of SARS-CoV-2 infection: A multi-centre, double blind, placebo-controlled, randomised trial. *J Clin Virol.* 2022; 155: 105248. doi: 10.1016/j.jcv.2022.105248. [↑](#footnote-ref-10)
11. Chandler DP, Wagnon CA, Bolton H Jr. Reverse transcriptase (RT) inhibition of PCR at low concentrations of template and its implications for quantitative RT-PCR. Appl *Environ Microbiol.* 1998; 64(2): 669-77. doi: 10.1128/AEM.64.2.669-677.1998. [↑](#footnote-ref-11)
12. Akerström S, Mousavi-Jazi M, Klingström J, Leijon M, Lundkvist A, Mirazimi A. Nitric oxide inhibits the replication cycle of severe acute respiratory syndrome coronavirus*. J Virol.* 2005;79(3):1966-9. doi: 10.1128/JVI.79.3.1966-1969.2005. [↑](#footnote-ref-12)
13. Laurie C, El-Zein M, Franco EL, Coutlée F. Assessment of the possible inhibitory effect of carrageenan in human papillomavirus DNA testing by polymerase chain reaction amplification. *J Med Virol.* 2021; 93(11): 6408-6411. doi: 10.1002/jmv.26963. [↑](#footnote-ref-13)
14. Ribeiro LS. Is there a safe way to remove carrageenan from RNA samples? Research Gate, Asked 18th Apr 2013.<https://www.researchgate.net/post/Is_there_a_safe_way_to_remove_carrageenan_from_RNA_samples> [Accessed 10.12.2022] [↑](#footnote-ref-14)
15. Tsang NNY, So HC, Cowling BJ, Leung GM, Ip DKM. Performance of saline and water gargling for SARS-CoV-2 reverse transcriptase PCR testing: a systematic review and meta-analysis. *Eur Respir Rev.* 2022; 20; 31(165): 220014. doi: 10.1183/16000617.0014-2022. [↑](#footnote-ref-15)
16. Seikai T, Takada A, Hasebe A, Kajihara M, et al. Gargling with povidone iodine has a short-term inhibitory effect on SARS-CoV-2 in patients with COVID-19. *J Hosp Infect.* 2022; 123:179-81. doi: 10.1016/j.jhin.2022.01.001. [↑](#footnote-ref-16)
17. Zou R, Peng L, Shu D, hao L, Lan J, Tan G, *et al.* Antiviral Efficacy and Safety of Molnupiravir Against Omicron Variant Infection: A Randomized Controlled Clinical Trial. *Front Pharmacol.* 2022; 13:939573. doi: 10.3389/fphar.2022.939573. [↑](#footnote-ref-17)
18. Thibon C, Vecellio L, Dubus JC, Kabamba B, Reychler G. Nebulization and COVID-19: Is the risk of spread actual? *Respir Med.* 2022; 197: 106854. doi: 10.1016/j.rmed.2022.106854. [↑](#footnote-ref-18)
19. Gandhi K, Paczkowski F, Sowerby L. Nasal irrigation to prevent and treat viral upper respiratory tract infections during the COVID-19 pandemic. *Otolaryngol Head Neck Surg.* 2022; 167(1 suppl): P285 [↑](#footnote-ref-19)
20. Burton MJ, Clarkson JE, Goulao B, et al. Antimicrobial mouthwashes (gargling) and nasal sprays administered to patients with suspected or confirmed COVID-19 infection to improve patient outcomes and to protect healthcare workers treating them. Cochrane Database Syst Rev. 2020; 9(9): CD013627. doi: 10.1002/14651858.CD013627 [↑](#footnote-ref-20)
21. Webster KE, O'Byrne L, MacKeith S, Philpott C, Hopkins C, Burton MJ. Interventions for the prevention of persistent post-COVID-19 olfactory dysfunction. *Cochrane Database Syst Rev.* 2022; 9(9): CD013877. doi: 10.1002/14651858.CD013877. [↑](#footnote-ref-21)
22. Ebell MH, Siwek J, Weiss BD, et al.. Strength of recommendation taxonomy (SORT): a patient-centered approach to grading evidence in the medical literature. *Am Fam Physician*. 2004; 69(3): 548-56. [↑](#footnote-ref-22)
23. Khan FR, Kazmi SMR, Iqbal NT, Iqbal J, Ali ST, Abbas SA. A quadruple blind randomised controlled trial of gargling agents in reducing intraoral viral load among hospitalised COVID-19 patients: A structured summary of a study protocol for a randomised controlled trial. Trials. 2020 Sep 14;21(1):785. doi: 10.1186/s13063-020-04634-2. PMID: 32928313; PMCID: PMC7487448 [↑](#footnote-ref-23)
24. Sheikh A, Ramalingham S. ELVIS COVID-19 Study. Could a simple saltwater solution lessen symptoms of COVID-19 and help people get better faster? <https://www.ed.ac.uk/usher/research/projects/elvis-covid-19> and <http://www.elvisstudy.com/> [Accessed 10.12.2022] [↑](#footnote-ref-24)
25. American Academy of Pediatrics. Classifying Recommendations for Clinical Practice Guidelines. *Pediatrics.* 2004; 114 (3): 874–877. https://doi.org/10.1542/peds.2004-1260 [↑](#footnote-ref-25)
26. Wise SK, Lin SY, Toskala E et al. International Consensus Statement on Allergy and Rhinology. *Allergic Rhinitis. Int Forum Allergy Rhinol*. 2018; 8(2): 108-352. https://doi:10.1002/alr.22073 [↑](#footnote-ref-26)
